# Understanding barriers to healthcare and harm reduction services among women who inject drugs in Nigeria: A qualitative assessment

**DOI:** 10.1371/journal.pgph.0007008

**Published:** 2026-08-13

**Authors:** Aisha Nantim Dadi, Olujide Arije, Kene Eruchalu, Jennifer Anyanti, Omokhudu Idogho, Yamen Okonkwo, Peace Ikani

**Affiliations:** 1 Society for Family Health, Abuja, Nigeria; 2 Institute of Public Health, College of Health Sciences, Obafemi Awolowo University, Ile-Ife, Nigeria; PLOS: Public Library of Science, UNITED STATES OF AMERICA

## Abstract

Women who inject drugs (WID) face barriers to healthcare, harm reduction, and social support, alongside stigma, violence, and legal risk. Evidence from Nigeria remains limited. This study explored how WID experience access to healthcare and harm reduction services and examined the social and structural conditions shaping that access. We used a phenomenological qualitative design in three Nigerian states: Abia, Gombe, and Oyo. Data were collected through 24 key informant interviews and six focus group discussions (FGDs) with WID (10 participants per group). Interview guides explored service availability, preferred access points, perceived quality of care, sexual and reproductive health needs, safety and violence, and barriers to service use. Data were audio-recorded, transcribed, de-identified, and analyzed thematically using ATLAS.ti 24. Four themes were identified: patterns of women’s injecting drug use, access to healthcare and harm reduction services, experiences of gender-based violence, and social and structural barriers. Sterile needles and syringes, HIV and sexually transmitted infection (STI) testing, and some family planning services were reported to be more consistently available through one-stop shops and outreach services, while antenatal care and prevention of mother-to-child transmission (PMTCT) services were mainly accessed through formal health facilities and were less frequently used due to stigma and fear of disclosure. Access through informal drug-use locations (“bunks”) and peer networks was frequently preferred due to convenience, anonymity, and reduced stigma. Improving access for WID requires integration of sexual and reproductive health services within harm reduction platforms, expansion of community-based and outreach delivery models, strengthening of trauma-informed care within health facilities, and establishment of safe and confidential referral systems that reduce exposure to stigma and law enforcement risk.

## 1. Introduction

Women who inject drugs (WID) face important health risks, including HIV, hepatitis C, and other blood-borne infections associated with unsafe injecting practices [1]. Their vulnerability is also shaped by gendered stigma, violence, unequal power relations, and unmet sexual and reproductive health needs [2]. Moreover, the gender-specific needs of WID, such as reproductive health services, are frequently overlooked in traditional drug treatment and harm reduction programs [3]. Beyond the health-related risks, WID are often subjected to gender-based violence, which significantly impacts their ability to seek and receive medical and social support. They are also exposed to physical, sexual, emotional, and psychological violence within intimate, community, and institutional settings [4; 5]. WID also face an elevated risk of intimate partner violence, often driven by socio-economic disadvantages and housing instability. Studies have shown that women who use drugs often do so in the context of intimate relationships, where violence is pervasive and associated with increased sexual risk behaviors [6].

The stigma attached to women’s drug use contributes to a cycle of exclusion that limits access to healthcare, legal support, and social services. In many settings, women who use drugs face stronger social judgment than men, with resulting concealment, delayed care-seeking, and weak social support [2; 7]. This gendered stigma also contributes to the poor fit of many existing drug-related services for women’s health and social needs. Additionally, the societal stigma associated with female drug use frequently leads to a lack of tailored health care and support services that address the specific needs of WID [8].

WID in Nigeria frequently experience unemployment and limited access to medications for opioid use disorder, factors that exacerbate their vulnerability. The United Nations Office on Drugs and Crime reported that 1 in 4 persons who use in Nigeria are women, and many face significant barriers to accessing treatment, including stigma and inadequate service provision [9]. In Northern Nigeria, cultural factors such as early marriage and gender inequality have been linked to increasing prescription drug misuse among women. A systematic review identified predictors of prescription drug abuse in this region, including unemployment, insurgency, early marriage, and lack of education [10]. These socio-economic and cultural barriers highlight the necessity for gender-sensitive drug policies and rehabilitation programs tailored to the needs of WID. Addressing these challenges requires comprehensive strategies that consider the unique experiences of women in different regions of Nigeria.

Harm reduction encompasses strategies aimed at minimizing the adverse health, social, and legal consequences associated with drug use, particularly among people who inject drugs [11]. These strategies focus on providing safer alternatives and treatment options to reduce the risks of HIV and other infections among people who inject drugs. For instance, needle and syringe programs and opioid substitution therapy (OST) have been effective in decreasing HIV transmission rates [12]. Among WID in Nigeria, injected substances reported in local sources include pentazocine, tramadol (including melted formulations), heroin, and cocaine [13]. Opioid agonist treatment (methadone, buprenorphine) remains limited relative to need, and needle/syringe provision occurs mainly through implementing-partner platforms (e.g., one-stop shops and outreach) rather than universal public coverage [14; 13].

Despite global evidence supporting the effectiveness of harm reduction, punitive drug laws and a lack of supportive policies impede progress in Nigeria. The criminalization of drug use and enforcement-related punitive measures have led to unintended consequences, such as increased HIV prevalence among people who inject drugs—from 3.4% in 2014 to 10.9% in 2020 [12]. These legal frameworks exacerbate stigma surrounding drug use, discouraging individuals from seeking help and increasing health risks, particularly among vulnerable groups such as women who inject drugs [12,15]. Additionally, Nigeria’s current drug policies lack effective public education components, which could play a significant role in mitigating drug-related harms [12].

Nigeria’s reliance on punitive drug policies has proven ineffective, necessitating a shift towards noncustodial approaches that incorporate social marketing strategies to educate and prevent drug use among youth [16]. While harm reduction strategies are essential, there is a concern that prioritizing them could inadvertently normalize drug use, potentially leading to increased consumption among at-risk populations. Understanding the specific needs and challenges of WID in Nigeria is crucial for developing effective, gender-sensitive interventions that address both health and social inequities. This study aimed to explore how women who inject drugs experience access to healthcare and harm reduction services in selected Nigerian settings, and how social and structural conditions shape those experiences. Specifically, the study explored reported patterns of women’s injecting drug use in the study settings, perceived access points and availability of healthcare and harm reduction services, experiences of violence and help-seeking as they related to safety and service use, and the broader social and structural barriers that affect access to care.

## 2. Methodology

### 2.1. Ethical considerations

The study received ethical approval from the institutional review board of the National Institute of Medical Research with the registration number NIMR/IRB/23/049. Written informed consent was obtained from all participants, ensuring compliance with ethical standards for research involving human subjects. The study adhered to the principles of confidentiality, anonymity, and voluntariness throughout the data collection and analysis phases.

### 2.2. Study design

This research employed a phenomenological qualitative design to explore the accessibility and utilization of health services by WID in Nigeria. The study integrated key informant interviews (KIIs) with key stakeholders and focus group discussions (FGDs) with WID.

### 2.3. Study setting

The study was conducted in three Nigerian states: Abia, Gombe, and Oyo. These locations were chosen based on their involvement in the Needle and Syringe Program (NSP), a critical component of the broader Optimizing HIV Investment Impact grant supported by the Global Fund, with the Society for Family Health as one of the principal implementers. The SFH’s operational presence in these states, particularly through the one stop shop (OSS) facilities, played a pivotal role in their selection. Initiated in July 2020, the NSP pilot aimed to address the needs of people who inject drugs, leveraging the findings from a key population size estimate conducted in 2018. These estimates highlighted Abia, Gombe, and Oyo as having significant prevalence rates of people who inject drugs among ten evaluated states, which also included Anambra, Edo, Enugu, Imo, Kaduna, Kano, and Taraba. The selection of these three states for the pilot program was purposive, targeting regions across different geopolitical zones to effectively gauge and respond to the health needs of the people who inject drugs within a diverse national context.

### 2.4. Study population and sampling

The study’s participants were drawn from a diverse group of stakeholders involved in drug use management and health service provision for WID. Key informant interviews targeted agencies interfacing routinely with WID: Harm Reduction Technical Working Group, State Agency for the Control of AIDS, Police Action Committee on AIDS, National Drug Law Enforcement Agency, key population secretariat, Association of Women Living with HIV/AIDS in Nigeria, Legal Aid Council, and the National Human Rights Commission. However, no clinicians were interviewed. This design prioritized lived experience and program/structural views aligned with study objectives. Box 1 below briefly describes the agencies/organization/group from which the stakeholders were drawn from. No financial incentives were provided.

**Box 1. Description of agencies/organization/group from which the stakeholders were drawn**

1. **National Human Rights Commission (NHRC):** A government agency responsible for promoting and protecting human rights, including addressing discrimination and violations related to HIV/AIDS.
2. **Association of Women Living with HIV/AIDS in Nigeria (ASHWAN):** A support and advocacy group for women living with HIV/AIDS, focusing on empowerment, healthcare access, and rights protection.
3. **Key population individuals:** Groups at higher risk of HIV infection, such as sex workers, people who inject drugs, men who have sex with men, and transgender individuals, often facing stigma and legal challenges.
4. **Legal Aid Council/ Ministry of Justice:** Provides legal support and representation for individuals facing discrimination, human rights violations, or legal issues related to HIV/AIDS.
5. **National Drug Law Enforcement Agency (NDLEA):** A government agency responsible for drug control policies, including interventions related to drug use and harm reduction for people who inject drugs, a key population in HIV prevention.
6. **Nigeria Police Action Committee on AIDS (PACA):** A unit within the police force focused on integrating HIV/AIDS response into law enforcement policies and reducing stigma against affected individuals.
7. **State AIDS Control of Agency (SACA)**: A state-level body coordinating HIV/AIDS prevention, treatment, and response programs in alignment with national policies.
8. **State AIDS and STIs Control Program (SASCP**): A public health program under the state health ministry responsible for managing and implementing HIV/AIDS and sexually transmitted infection (STI) control efforts.

We used purposive sampling to recruit 60 WID across urban and rural communities for the FGD. Recruitment took place directly at hotspots where WID congregate some of whom are part of ongoing interventions. An outreach worker who routinely engages with WID at the hotspots approached potential participants face-to-face, described the study, and obtained initial agreement. Phone contacts were collected, and reminders were provided one day before each session. Over 20 women were initially contacted per site; those available on scheduled dates were transported to the venues for the FGD session. Inclusion criteria were: female; age 18–49 years; currently injects drugs; physically able to participate; provides verbal informed consent; agrees to audio recording. Individuals who were unable to provide informed consent because of cognitive impairment or acute intoxication at the time of recruitment were excluded. Six FGDs were conducted (2 per state, 10 participants per group). Recruitment and enrollment were completed within three weeks, enabled by close social networks and peer referral. The sample is not statistically representative of the region; it reflects WID within the selected bunks. Also, FGD participants were recruited via multiple entry points (bunks, hotspots, peer referral) and were interviewed by independent research staff who were not part of service delivery. Reported experiences therefore reflect both program-linked and community settings.

'Bunk' in the context of drug-using communities in Nigeria designates not only the geographical localization of living spaces predominantly occupied by individuals engaged in drug injection practices but also implies a structured hierarchy within these areas. This hierarchical structure manifests in the organization and dynamics of the community, influencing aspects such as control over drug supply channels, allocation of resources, and decision-making processes. Individuals or groups at higher levels of this hierarchy may exert influence or control over those at lower levels, affecting access to drugs, living conditions, and safety within the community. This hierarchical organization can further exacerbate vulnerabilities among community members, leading to disparities in health outcomes, security, and social support. The term bunk thus encompasses the complexities of spatial, social, and economic factors that characterize the living environments of drug-using populations in Nigeria, highlighting both the physical geography and the embedded social structures.

Detailed demographic data were intentionally limited to reduce re-identification risk. For women in the FGDs, adult eligibility and current injecting status were confirmed for study participation. Pregnancy- and HIV-related issues are presented in the results only where participants discussed them in relation to service access and care needs. For key informants, only institutional role and organizational affiliation were reported.

Interviews and FGDs were conducted by female research assistants contracted for the study. All held at least a university degree and prior qualitative experience. A one-day refresher covered research ethics, interviewing techniques, and handling sensitive topics. Interviewers had no prior relationships with participants and were independent of recruitment locations. Outreach workers (implementing organization staff) handled mobilization and transport only; they did not conduct interviews. Purposive recruitment was used across states for FGD participants; refusals/withdrawals were not systematically tracked, although study facilitators reported occasional same-day no-shows that were replaced opportunistically, and no repeat interviews were conducted. Participants did not receive financial compensation.

Table 1 below shows the different categories of study participants. Participants received refreshments during sessions and transport money for return travel (for those who incurred transportation cost), per ethics approvals.

**Table 1:** Categories of study participants and number of interviews or discussion sessions.

Category	Type	Number of interviews/sessions
National Human Rights Commission representative	KII	3
Association of Women Living with HIV/AIDS in Nigeria representative	KII	3
Key population individuals	KII	3
Legal Aid Council/ Ministry of Justice representative	KII	3
National Drug Law Enforcement Agency representative	KII	3
Nigeria Police Action Committee on AIDS representative	KII	3
State Agency for the Control of AIDS representative	KII	3
State AIDS and STIs Control Program representative	KII	3
Women who inject drugs	FGD	6

### 2.5. Study instrument

The interview and FGD guides used explored broad domains related to injecting practices, access points for health and harm reduction services, perceived service quality, sexual and reproductive health needs, safety and violence, help-seeking pathways, referral systems, and barriers to service use. The KIIs focused on program and institutional perspectives, while the FGDs focused on lived experience, everyday service access, and perceived risks in care-seeking.

### 2.6. Data collection

Participant recruitment occurred between October 9–31, 2023 while data collection was conducted from November 13–30, 2023. A total of 24 KIIs were conducted across eight stakeholder categories, with three interviews per category. The interviews captured programmatic, policy, legal, human rights, and community perspectives. Topics included available services, referral pathways, service gaps, drug use patterns, and barriers affecting women who inject drugs. Six FGDs were conducted with women who inject drugs, with two FGDs per state and 10 participants per group. These discussions captured shared experiences, service preferences, group norms, and daily barriers. The approach ensured inclusion of perspectives from women with lived experience.

Rather than determining saturation through iterative assessment during data collection, sample adequacy in this study was informed by a predetermined data sources and maximum variation strategy. Before data collection, we specified the number of states, the number of FGDs per state, and the key stakeholder groups relevant to women who inject drugs, so that the sampling frame would capture the anticipated range of variation in experiences and perspectives across settings. This a priori approach — sometimes referred to as an information power or sampling adequacy strategy — was designed to ensure adequate coverage of relevant sites and stakeholder groups rather than to confirm saturation empirically through concurrent analysis [17].

All discussions were audio-recorded with prior participant consent and conducted in private settings to ensure confidentiality. Interviews were conducted in English, Nigerian Pidgin, Yoruba, and Hausa based on participant preference. Bilingual facilitators and note-takers supported mixed-language sessions. Session durations were not formally recorded; however, most FGDs lasted approximately 60–90 minutes and KIIs 30–60 minutes.

### 2.7. Data management and analysis

Audio recordings were transcribed verbatim and translated into English where necessary. Names of persons, facilities, and organizations were removed or replaced with neutral descriptors after coding was completed. Analysis followed thematic content analysis using a modified in-vivo approach [18] in ATALS.ti 24. An initial coding framework was developed from the interview guides and was then refined iteratively as coding proceeded. This allowed both pre-identified domains and emergent issues from the data to be captured. A group of three external qualitative analysts conducted first-cycle coding, after which the lead researcher reviewed and refined the coding structure. Themes were developed through comparison across KIIs and FGDs and across states, with attention to both convergence and variation in accounts.

Because the FGD and KII guides covered different thematic areas, their resulting data were analyzed separately. The analysis outputs are presented in S1 Data, organized thematically in Excel format. Each sheet within S1 Data corresponds to one theme. Within each sheet, data are organized by theme, subtheme, sub-subtheme, and illustrative quotation. All personally identifying information has been removed from the dataset during review.

## 3. Results

The findings from this study are divided into broad areas of thematic emphasis as follows: characterization of injection drug use among women, health care services for WID, gender-based violence and WID, and social services for WID. Verbatim quotations from FGD participants represent lived experiences of the women, while other quotations represent the perspective of the various service providers and stakeholders interviewed.

### 3.1. Characterization of injection drug use among women

#### 3.1.1. Characteristics of women who inject drugs.

Stakeholders described three recurring and overlapping groups of women who inject drugs in the study settings: younger women, women who remained socially unnoticed, and women living alone in socially marginalized environments. These are descriptive groupings rather than fixed categories. Stakeholders linked these positions to reduced visibility, stigma, and limited support, while women in FGDs also described social neglect, concealment, and isolation in ways that broadly aligned with these accounts.

#### 3.1.2. Types of drugs used.

Stakeholders interviewed reported a wide range of injectable substances used by persons who use drug, especially women. Cocaine was one of the most frequently mentioned drugs. An interviewee from Oyo SACA said simply, *“Cocaine”*, while another added, *“Cocaine and heroin, though they are expensive, but commonly used by them... most of the women are not using their money to buy the drugs, men can give it to them.”* A legal aid participant from Gombe remarked, *“I’m very sure it could be found with women who inject drugs.”* Heroin was also widely mentioned. Another stakeholder stated, *“Heroin is commonly used here”*, while others listed it alongside cocaine and other opioids. Tramadol featured prominently, with reports of it being melted for injection. One participant said, *“sometimes the Tramadol can be melted. When you melt it, then you now draw a line… That’s when you inject it” (ASHWAN/Abia).”*

Pentazocine (often called “Penta”) was described as the most commonly injected drug in some areas. A stakeholder explained, *“I think the drug they normally inject here is Pentazocine,”* and another stated, *“We find young women, married women who inject themselves with drugs like pentazocine.”* Other reported substances included *“Heroine, Ketamine, and Cocaine, and SK(*SK most commonly refers to skunk—a high-potency form of cannabis (aka “loud,” “Arizona”). It’s typically smoked, not injected*)”.* Stakeholders also mentioned newer or local substances like ‘*Mkpụ*r*ụ Mmiri’*(Mkpụrụ Mmiri is an Igbo (one of the major Nigerian languages) slang term for methamphetamine (crystal meth). The phrase literally translates to “seed of water” and refers to the drug’s appearance as clear, crystalline chunks.) and concoctions with unknown ingredients. One person observed, *“Some make concoction that change colour to zobo* [a common local non-alcoholic drink]*... they have already cooked it and add a colour to it.”* Another noted, *“They usually start with pain reliever…, then go to sedative... and a mixture of other drugs that you can’t even put a name to.”* The findings highlight a complex mix of pharmaceutical opioids, street drugs, and improvised substances being injected, often without clear knowledge of the chemical content.

#### 3.1.3. Methods of injection.

According to some of the FGD participants, services such as the provision of needles and syringes is consistently made accessible to them at affordable cost and desirable quantities. They reported obtaining needles and syringes from one-stop shop distribution and outreach, bunk suppliers, and retail pharmacies; a minority sourced from peers. Formal exchange/return was described at one-stop shops and in some bunk settings. A stakeholder in Gombe shed light on the tendency of individuals to seek privacy indoors such as homes for drug sharing and injection. The participant stated, “*They normally hide inside one room to share a needle to do the injection*” (Gombe/Key population representative). Also, the injection of Kolos—a substance similar to Indian hemp, was described as a particularly hazardous trend among WID by participants from ASHWAN in Abia. The method of injection for Kolos involves the boiling and subsequent injection of the substance. Additionally, a participant from Abia underscored the practice of melting tramadol for injection, “*Sometimes the Tramadol can be melted. When you melt it, then you now draw a line… That’s when you inject it*” (Abia/Key population representative). The predominant method of drug injection reported across the study locations is intravenous, wherein arms or legs are tied to reveal veins for injection. A participant from Gombe’s SACA noted the unsafe practice of tying hands during injection, leading to injuries, which on the long run may potentially require amputations. Another alarming practice noted by the NDLEA in Abia involves the injection of one’s blood by the WID in desperation for intoxication. He said: *“…you have someone showing you he doesn’t have money to buy. The next thing you see is to draw out their blood and inject it on the other person.”*

### 3.2. Health care services for WID

#### 3.2.1. Place of health care services access.

We found that WIDs accessed care across multiple venues—OSS, public PHC/hospital services, private clinics, community chemists and retail pharmacies, and via mobile/outreach (including episodic service delivery in bunks). While bunks were familiar and perceived as discreet, formal clinical care remained anchored in OSS and health-facility settings. FGD participants from Oyo expressed a preference for receiving healthcare at the '*bunk'* due to its familiarity, perceived security, and affordability. One participant stated that:

“I see OSS as one stop shop where I can easily come and make my complaints and take the medication I need and go my way easily” (FGD participant/Oyo)

“I prefer going to the bunk because that’s where I am used to right from time,...I like that place more than any other place. I don’t know about OSS or any other place.” (FGD participant/Oyo)

When asked for why of the choice of healthcare service location, responses varied. Free access to health-related services emerged as a major reason.

“It’s not that expensive, I don’t know, it happens sometimes he just gives us free at times” (FGD participant/Abia)

“No, they don’t take money. They do it free of charge.” (FGD participant/Abia)

Other reasons include medication availability, proximity, treatment effectiveness, and the professionalism of service providers. Participants also highlighted the convenience of accessing healthcare services at familiar locations like the bunk, where they feel comfortable and secure. For instance, the OSS was favored by some WID for its conducive, calm, and discreet environment, with some stating,

“… this is the place that I know that when I am coming to, no police will follow me at my back. I come straight, collect it and leave and I do not know what happens around. So here … for me is safe” (FGD participant/Abia).

Other reasons given include short waiting time (“*You are being attended to as soon as possible*” the proximity and the quality of service offered *(“They give us better services*”*).* Additionally, participants described pharmacy purchase when program supplies were depleted: *“if the one given to us finishes, we used to go to the pharmacy and buy another one” (FGD participant/Gombe).”*

#### 3.2.2. Needles and syringes.

Participants described consistently available sterile injecting equipment delivered through one-stop shops and community outreach, with additional access at bunk-based activities and, at times, through retail medicine stores/patent and proprietary medicine vendors. Distribution was portrayed as routine and oriented toward single-use to reduce sharing, and formal needle–syringe exchange/return was linked to OSS operations and reported during some bunk-side engagements. A smaller group still relied on peers’ equipment when supplies were unavailable or unaffordable. The following are quotes for study participants:

“Yes, there is an intervention, that’s the first thing I will talk about the intervention … in Gombe state through the [implementing organization]. I want to applaud them for that because the intervention is that they bring a syringe and needle which means only one person can use it…” (FGD participant/Gombe).

“The cost I purchased the 2ml size is ₦100... I prefer to get it once and stock at home” (FGD participant/Oyo)

“They will rather go medicine store (patent medicine vendors) and get services from unqualified nurse…” (SASCP/Gombe)

“Before the provision of this needle and syringe some of us we do share needles… if my friend injects, after her, I will also inject with the same needle… because I don’t have money to buy my own.” (ASHWAN/Abia)

On whether WID reuse or share syringes, a participant from Gombe stated, “*Sometimes we make use of that same syringe and needle for many times until is no more functioning*” (FGD participant/Gombe). A participant from Abia said, “*It’s only one they use and they dispose of it after using*” (FGD participant/Abia), another stressed that “*it’s not what should be used twice, once used, dispose of*” (FGD participant/Abia), and another replied a firm, “*No ooo*” when asked if they give another person to use after finishing (FGD participant/Abia). A participant from Oyo stated that

“I usually get my needle and syringe from the bunk as I get my drugs to take home because without it, how will I inject my drugs? It seems there are some people who supply the bunks with needles and syringes. Immediately after use, we don’t reuse it and return the used needle and syringe in a trash box provided in the bunk but I don’t know how it’s later disposed of. They give us needles and syringes for every purchase since we can’t use one needle twice” (FGD participant/Oyo)

Lastly, various disposal practices were mentioned by the participants including toilet, bush, and trashcan disposal. A participant stated, “*I bring it back to the OSS*” (FGD participant/Abia) while another said, “*Sometimes OSS used to give us sharp box when its full, we return then they give us another one*” (FGD participant/Gombe).

#### 3.2.3. Healthcare packages for WID.

WID are reported to receive treatment for various illnesses, including HIV, Hepatitis, and STIs. Testing precedes treatment, with STI tests being common, as well as the administration or prescription of necessary drugs post-laboratory tests. Participants attest to the availability of testing and drug provision:

“Sometimes they used to run a test for us for any disease they found if they have the drugs, they will give it out and if they don’t have, they will prescribe some drugs for you to be using” (FGD participant/Gombe State)

“They are ready to and capable of running any [lab] test you want them to do for you. They do all the tests for you when you get there, I just did my hepatitis test there a few days ago” (FGD participant/Oyo State)

Additionally, personal hygiene products like sanitary pads and soaps were made available for the WID while pregnant. A participant from Abia stated, “*Sanitary wear, for people that are pregnant, sanitary use… Soap, all these our toilet uses, understand? For people that are pregnant, because not all can afford*” (FGD participant/Abia). It should be noted that the provision of some of these services were within specific intervention context. WID also often have family planning methods and counselling available to them by their healthcare providers to WID to address the risk of unwanted pregnancies among. A participant from Gombe affirmed that they sometimes get “*family planning, sometimes drugs*” (FGD participant/Gombe State), while another said, “*Yes, I told him and he advises me on the best family planning method for me*” (FGD participant/Oyo) when asked if she got counselling for family planning awareness.

Also, healthcare providers in the study area are said to help in the facilitation of preventing mother-to-child transmission of HIV through the provision of anti-retroviral drugs, and monitoring of HIV-positive pregnant WID and breastfeeding practices. A participant stated,

“It is possible you have HIV and you are pregnant so they will look for a way to give you drugs so that the child will not be affected. After delivery, they also make sure that they monitor you and the way you breastfeed the child” (FGD participant/Gombe).

Antenatal care services are also accessible to WID as affirmed by the response from a participant from Abia*, “It’s antenatal… Yes, I have accessed it before” (FGD participant/Abia).*

In addition, incentives and free services were said to be provided for WID to encourage them to access services like screening/testing services. One participant from Gombe said,

“There was a time that to draw the attention of people to come for this check-up they used to say that there is a gift they will give out to people … so this makes people come out for the test” (FGD participant/Gombe).

Another participant from Abia affirmed this saying, “*They give us money when we go to their hospital… The other places charge us, but you people do not charge us*” (FGD participant/Abia). Some responses from the participant shows that the provision of free healthcare services ensures accessibility for WID. For instance, an FGD participant from Gombe said that. “*Before this program, we used to buy it* for ₦*50 and above but now sometimes we give them for free*” (FGD participant/Gombe).

Access to antenatal care (ANC) was discussed across Abia, Oyo, and Gombe: several participants described prior ANC use and prevention-of-mother-to-child-transmission (PMTCT) support— *“It’s antenatal… Yes, I have accessed it before”* (FGD participant/Abia); *“it is possible you have HIV and you are pregnant so they will look for a way to be giving you drugs so that the child will not be affected… and after delivery they also make sure that they monitor you”* (FGD participant/Gombe). At the same time, others linked inconsistent ANC to stigma and fear of disclosure— *“when they are pregnant… due to that stigmatization, they don’t go to hospital… They start to… shade themselves away from the public”* (FGD participant/Abia); some *“don’t go to antenatal”* at all (FGD participant/Abia), and negative facility encounters reinforced avoidance— *“the way they treated her that day… anytime I have problem… I don’t bother to go”* (FGD participant/Abia). In Oyo, women described concealing drug use when seeking care— *“I wouldn’t let anyone know I’m a WID at all… because of the discrimination and stigmatization”* (FGD participant/Oyo)—underscoring how stigma undermines consistent ANC uptake despite reported availability.

#### 3.2.4. Perception of WID about current service offered.

Many participants expressed satisfaction with the services received, citing factors such as quality, accessibility, and the attitude of service providers. A participant from Oyo highlighted the quality of service as a key factor contributing to her satisfaction: “*If you get to the pharmacy, I get mine from, they will give you the best service you can ever think of*” (FGD participant/Oyo). She emphasized the professionalism and safety of the pharmacy, attributing her confidence in the services to the high standards maintained. Echoing this sentiment, another participant from Oyo emphasized the importance of quality over convenience:

“If you consider the advantages and services, you will get, and in a case where I have an infection if I compare the medications, they will give me to the money I’ll spend, it’s almost the same thing and also the cost of transportation is so expensive at the moment but you have to consider your health” (FGD participant/Oyo).

Similarly, a participant from Abia expressed satisfaction with the quality of condoms provided: “*Yes. It’s because the condom is original. If you put it, it does not break the way others break*” (FGD participant/Abia). A participant from Oyo appreciated the respectful and approachable demeanor of the staff: *“I prefer the way they attend to us there, because it is ok. They do not embarrass us, they are composed and nice people there, both male and female”* (FGD participant/Oyo).

However, not all participants shared this positive perception. One participant from Abia voiced dissatisfaction with the quality of service received at a particular hospital: “*I go to* [hospital name] *but I don’t really like their quality service*” (FGD participant/Abia).

#### 3.2.5. Client outreach.

Understanding how various service providers reach WID is essential for effectively providing them with necessary services. Stakeholders in the study area employed various methods, including outreach, referrals, walk-ins, arrests, snowballing, and communication channels like phone calls.

**Outreach:** Many stakeholders engage in community outreach to connect with WID who may be hesitant to seek services due to fear of discrimination or arrest. By conducting awareness programs and offering testing services in communities, stakeholders build trust and encourage WID to approach them. As one stakeholder from Abia noted, “*Sometimes, people who know about us... become attached to us. Some of them even come to our office... when they feel that they don’t have any place to go*” (ASHWAN/Abia).

**Referrals:** Stakeholders also receive clients through referrals from various sources, including police, lawyers, arresting authorities, and community-based organizations (CBOs). Referrals may come from individuals seeking legal assistance or intervention in drug-related cases. According to a stakeholder from Gombe, “*We get clients by referrals from either the arresting authority... or from CBOs... we have some affiliation with*” (Legal Aid/Min of Justice/Gombe). Family members, friends and even WID among themselves sometimes refer other WID for counselling and support services. According to one participant who is a WID herself, “*Some of them, like this my brothel, I have about 12 persons and about seven or eight of them do take drugs. Some of them have been brought into the bunk by me to stay with me*” (ASHWAN/Abia).

**Walk-ins:** Some stakeholders receive clients who voluntarily visit their offices without prior outreach or referrals. These “walk-in” clients demonstrate initiative in seeking assistance. As one stakeholder from Abia mentioned, “*We don’t go out scouting for clients, they come to the office on their own*” (NHRC/Abia).

**Arrests:** In certain cases, stakeholders, particularly law enforcement agencies, obtain clients through arrest operations targeting drug-related offences. This method involves apprehending individuals involved in drug trafficking or consumption. As stated by a stakeholder from Abia, “*Our client... can come sometimes through operation... we tend to know which ones are dealers and which ones are users*” (NDLEA/Abia).

**Snowballing:** Snowballing involves leveraging existing connections with WID to expand outreach efforts. Stakeholders rely on trusted contacts within WID communities to introduce them to others who may require services. This method facilitates access to hard-to-reach populations. A stakeholder from Oyo explained, “*We have been working with them for a while... if you have the contact of one of them, then you can get another... reaching them is not difficult at all*” (SASCP/Oyo).

**Communication Channels:** Additionally, stakeholders provide avenues for WID to reach out for assistance through communication channels such as phone calls, emails, or text messages. This allows WID to communicate their needs or seek guidance remotely. As stated by a stakeholder from Oyo, “*We have other means of receiving complaints, which could be through electronic means, via emails, text messages, even on a phone call*” (NHRC/Oyo).

Table 2 below summarizes services available to WID in the study areas.

**Table 2:** Reported availability of healthcare, harm reduction, and support services for women who inject drugs across study settings.

Service domain	Reported availability	Main access points reported	States/settings mentioned	Notes from participants and stakeholders
**Sterile needles and syringes**	Reported available, but not always consistent	One-stop shops, outreach, bunks, pharmacies/medicine stores	Abia, Gombe, Oyo	Some women still reported reuse or sharing when supplies or money were limited
**Needle return and sharps disposal**	Reported in some settings	One-stop shops, some bunks	Abia, Gombe, Oyo	Disposal also reportedly occurred in toilets, bushes, or trash when formal return systems were absent
**HIV, STI, and hepatitis testing**	Reported available	One-stop shops, hospitals/clinics	Mainly Gombe and Oyo; also discussed across sites	Testing often preceded treatment or referral
**Treatment or referral for HIV, STI, and hepatitis-related conditions**	Reported available within program-linked settings	One-stop shops, referral facilities, hospitals	Abia, Gombe, Oyo	Access depended partly on drug and commodity availability
**Family planning counselling and methods**	Reported available	One-stop shops and other providers	Gombe, Oyo	Discussed mainly by FGD participants
**Antenatal care and PMTCT support**	Reported available but inconsistently used	Hospitals/clinics, referrals	Abia, Gombe, Oyo	Use was reduced by stigma, fear of disclosure, and poor treatment at facilities
**Menstrual and personal hygiene supplies**	Reported in some intervention settings	Program-linked services	Abia	Described as context-specific rather than routine everywhere
**Gender-based violence (GBV) reporting and support**	Limited and inconsistent	One-stop shops, NHRC, church, police	Abia, Gombe, Oyo	Many women reported weak or absent support pathways
**Rehabilitation and follow-up services**	Major gap reported	Rehabilitation centers and referrals	Reported by stakeholders across states	Lack of centers, weak follow-up, and limited continuous counselling were noted
**Services for children of women who inject drugs**	Major gap reported	Not specifically described	Reported by stakeholders	Stakeholders requested inclusion of children in programming

*Note: WID = women who inject drugs; FGD = focus group discussion; STI = sexually transmitted infection; PMTCT = prevention of mother-to-child transmission; NHRC = National Human Rights Commission; OSS = one-stop shop.*

### 3.3. Gender-based violence (GBV) and WID

#### 3.3.1. Experience of GBV by WID.

Women injecting drugs face different types of abuse, including physical, emotional, psychological, and economic violence from intimate partners, community members, and even those from whom they seek assistance. These experiences include:

**Beating by partner:** Findings reveals instances of severe physical abuse inflicted by intimate partners, as exemplified by a participant from Oyo who recounted being beaten by her husband. Despite reporting the abuse to authorities, she faced challenges in resolving the issue, reflecting the pervasive nature of domestic violence. She expressed, “*My husband beat me up... When I reported to my family, we went to meet him... and we beat him up too... he was locked up... but when people started begging me... we settled amicably*” (FGD participant/Oyo).

**Risk of Fatal Retaliation:** The fear of reporting abuse is heightened by the perceived risk of retaliation. A participant from Abia expressed concerns about potentially fatal consequences if abuse is disclosed. The participant highlighted the precariousness of their situation, stating, “*Maybe the boyfriend is a bad person... If she reports, she may be killed... They may kill us*” (FGD participant/Abia).

**Disgraced and Disrespected:** WID endure public humiliation and disrespect, not only from intimate partners but also from members of their community and even vendors from whom they seek assistance. They face insults, ridicule, and exploitation, exacerbating their vulnerability. As expressed by participants from Gombe, “*Someone very close to you may even insult you... even in the community, you don’t have freedom, they will always disgrace you*” (FGD participant/Gombe).

However, not all participants had experienced GBV, as one from Abia noted, “*I’ve never had such experience before*” (FGD participant/Abia).

#### 3.3.2. Reporting of GBV.

Findings from the interviews showed that WID use different avenues for reporting instances of GBV, influenced by factors such as safety, trust, and perceived efficacy of reporting mechanisms. Some WID turn to human rights centers to report abuse, viewing them as allies in seeking justice and protection. These centers offer a sense of security and advocacy for WID facing GBV, as highlighted by a participant from Gombe who stated, “*They are places that we do take reports to... in the* (National) *Human Right Commission*” (FGD participant/Gombe). For some WID, spirituality serves as a sanctuary wherein they find solace in prayers and communion with God amidst experiences of violence. A participant expressed a sense of comfort in confiding their struggles to a higher power. As one participant from Abia shared, “*I have experienced violent attack before... but I only went to church to talk to God about it, no other place to go to report*” (FGD participant/Abia).

Despite being a traditional avenue for seeking justice, participants expressed disenchantment with the police force, citing instances of maltreatment and corruption. The prevalent perception is that police prioritize the interests of perpetrators over those of WID. One participant recounted being slapped and robbed by a police officer when attempting to report abuse:

“Like there’s this my girlfriend that got this money, I don’t know where she got it, so the men caught her and they used lighter and razor blade to cut and light her vagina. I was asking, even the police could not help. Even the hospital we took her to…, she was rejected. So, when such happens, no one comes to our rescue. Even our vendors don’t come to rescue us from danger. Police don’t even rescue us because the (drug) dealers settle them.” (FGD participant/Abia)

Additionally, in spite of the prevalence of GBV, many WID opt for silence and self-reliance due to perceived futility in reporting and fear of further victimization. They grapple with enduring abuse without recourse to formal support systems. As one participant lamented, “*We don’t report because they take us as useless human beings... even if you report, they won’t do anything*” (FGD participant/Gombe). However, some participants identify OSSs as safe spaces for reporting violence and accessing support services. When asked, “*If someone is violent towards you, like maybe your friend asks you to shut up and maybe defaults on his agreement with you, where will you go to report?*”, a participant from Abia responded, *“This is where I will come.”* referring to the OSS (FGD participant/Abia).

Notable gaps in services related to protection against GBV include reports of lack of support for sexual assault victims. A participant said, “*Anything we see, we face, we carry it on our own… We don’t have any help*” (FGD participant/Abia), another stated that they “*don’t have such services here, once the person is off, the person is off*” (FGD participant/Abia), while one participant from Oyo lamented, “*Even when we have females who are abused among us, there is no one to call for help*” (FGD participant/Oyo).

### 3.4. Barriers to services for WID

#### 3.4.1. Social and structural barriers.

Participants reported various barriers to accessing services, including high costs, long distances, discrimination, professionalism of service providers, and negative attitudes from health providers. The inflation-induced high cost of services hindered access, as mentioned by a participant from Gombe: “*the drugs before we used to buy it at the rate of ₦100 or* ₦*80 and now you can even go and get it at the rate of ₦1,000 this is a big challenge to us*” (FGD participant/Gombe). A participant from Abia cited long distances as barrier to accessing service points stating, “*Sometimes if I don’t have money for transport...I won’t be able to come*” (FGD participant/Abia). Discrimination and stigma from service providers and society also posed challenges. A participant from Gombe noted, “*when you go for care...they don’t accept us*” (FGD participant/Gombe), while another from Oyo mentioned, “*They shout at us at times, and when there is no money, they wouldn’t attend to us*” (FGD participant/Oyo).

#### 3.4.2. Arrest by security personnel.

Participants reported experiencing harassment, arbitrary arrests, and extortion by security agencies, particularly the police and NDLEA. During raids, participants felt targeted and stereotyped, as one from Oyo noted: *“The police always feel we are addicts...and we cannot do without taking our drugs”* (FGD participant/Oyo). Exorbitant bail amounts were also reported, with one participant from Abia stating, *“…If you don’t have ₦20,000 or more than that, my sister meet me in court”* (FGD participant/Abia). Some participants experienced even more severe exploitation, including demands for sexual favors, as a participant from Gombe shared: *“the female if you don’t have money to bail yourself, they ask to have sex with you”* (FGD participant/Gombe). The intensity of police raids was also highlighted, with a participant from Oyo describing: *“If you see the female police officers that come to bunk, you would be scared because if you see her chasing people, you won’t believe how swift they are” (*FGD participant/Oyo). Lastly, according to the participants, arrests occurred at joints/bunks, homes, and even on the road, with one participant from Gombe stating, “*there was a time they enter our room and arrest us*” (FGD participant/Gombe). Reasons for arrest included drug possession, being found in prohibited locations, and suspicion of prostitution, as mentioned by participants from Abia and Oyo.

#### 3.4.3. Gaps in services.

Stakeholders from various States highlighted several key areas where existing services fall short. One significant issue is the non-inclusion of WID children in existing programs, leaving them vulnerable to risks and societal challenges. A stakeholder from Abia emphasized the importance of considering the welfare of WID’ children, stating, “*While you’re programming for women who inject drugs, remember also to program for their children*” (SACA/Abia).

Additionally, there’s a lack of follow-up during rehabilitation, with agencies failing to monitor the progress of WID after they’ve been brought to rehabilitation centers. This neglect was criticized by a stakeholder from Abia who stressed the “*need for constant follow-up on these women*” (SASCP/Abia). Moreover, the absence of rehabilitation centers in certain regions exacerbates the problem, as observed in states like Gombe, where stakeholders have called for the establishment of such facilities to ensure proper monitoring and support for WID:

“...the government also need to do more, engage them if possible, rehabilitate then reintegrate them back into the society and if maybe empower them economically because if that is not achieved then definitely you know they will continue… Monetary empowerment and integrated back to the society and they are rehabilitated so the government need to come in an NGO also needs to come in to bridge this gap” (NHRC/Gombe)

Furthermore, poor funding poses a significant challenge to addressing the needs of WID. Stakeholders from Oyo highlighted the lack of funding for specific issues: “*There is no funding for Hepatitis B for now, but I know if there is funding for Hepatitis B, they will start referring them, and they will get better*” (ASHWAN/Oyo). Insufficient counselling services for WID, both during rehabilitation and afterwards, further compound the problem, as emphasized by a stakeholder from Abia: *“…there is a need to also improve counselling, counselling is very important, when they are properly counselled and counselled, they will be able to understand where they are and the disadvantages of injecting drugs…Proper counselling continuous counselling is very important”* (SASCP/Abia).

Additionally, poor community involvement and lack of support from religious and traditional leaders hinder the effectiveness of programs aimed at helping WID. A participant said:

“Like I mention before the barrier are that our religion leaders, tradition leaders need to come around to see to the health sector just like I said we need to accept the fact that our women are into drugs, our women are injecting drugs, and once we do that then we will begin to look at how do we go about it to help them because they need hey, they need support” (Gombe/SACA).

Stakeholders also highlighted the absence of programs specifically targeted at WID. According to a participant:

“Currently we have programs for the people who inject drugs, but there is none specifically for WID, and you know that these people have their own peculiarity. Being a woman, there would be some peculiar challenges, so there might be a need for us to have a program specifically targeted towards” (SASCP/Oyo).

Stigmatization by healthcare providers further complicate efforts to support WID

“And even when we go to receive treatment, they will say these ones are drug addicts. Hence, they don’t attend to us as they are supposed to. Like I said earlier, they regard us as outcasts wherever we go because of the drugs we take.” (ASHWAN/Abia).

Stringent government policies and poor recruitment strategies contribute to the difficulties in reaching and assisting WID. Stakeholders stressed the importance of relaxing policies to encourage WID to seek help and implementing effective recruitment methods to identify and support these individuals:

“It’s not a matter of shying away from it because we all know that whether we like it or not we have women who are injecting drugs, we have women who are taking drugs and it could be your wife, sisters you might never know but they are there. So the government, policy makers, we need to relax on some issue on policy so that these women and come out and get help. I think that government policy should relax a little to allow health sector to go out seek for this hot spot of these people, these women…” (SACA/Gombe).

However, a representative from Gombe/Legal Aid/Ministry of Justice points out the sensitivity in advocating for the rights of WID, where it is perceived as promoting drug use. She said:

“...when you are trying to sensitize and authority about the right or the wrong of people or women injecting drugs it seems like you are not, the cloud as you are promoting and you are facilitating such act so is very difficult and challenging to be frank” (Legal Aid/Min of Justice/Gombe).

### 3.5. Potential facilitators to improving healthcare access for women who inject drugs

Recommendations for addressing the needs of WID were proposed by the study participants. Firstly, employment emerges as a crucial focus area, with WID expressing a strong desire for job opportunities to help them stay focused and avoid involvement in drugs and crime. Stakeholders from Abia and Oyo emphasize the importance of government-led empowerment and job creation initiatives, as articulated by a participant from Abia who pleaded, “*Please government should come and rescue and secure us so we don’t become useless because this is not the way God has made us to be*” (FGD participant/Abia).

Secondly, the establishment of specialized health facilities tailored to the needs of WID is recommended to address the discrimination and stigmatization they face in accessing healthcare services. Participants advocate for dedicated centers where they can receive medical care without fear of discrimination, particularly important for pregnant WID or those with children. A participant from Oyo highlights this need, stating, “*We will appreciate it if you can support, we the WID. There are some of us who are pregnant or have kids and can’t visit the bunks or hospitals because of discrimination. But if there are centers that you can provide for us, and we are comfortable, it will help a lot*” (FGD participant/Oyo).

Lastly, ensuring accessibility to essential commodities such as needles, syringes, and condoms is crucial in preventing the spread of diseases and unwanted pregnancies among WID. Participants from Oyo stress the importance of having these commodities readily available and urge for increased efforts in ensuring their availability. One participant emphasized the challenges they face in accessing these commodities, stating, “*As regards the needle and syringe, most of the people that come to give us will say they don’t have or it’s finished. Sometimes they would say they don’t have condoms but they have lubricants only. So, they should put more effort in this for us*” (FGD participant/Oyo).

## 4. Discussions

In this study, we assessed the perspectives of stakeholders involved in interventions targeting WID within the context of ongoing interventions in selected states in Nigeria. The diversity in the pattern of drug use reported among WID including substances like cocaine, codeine-based drugs, heroin, and an array of locally concocted substances, mirrors global trends highlighting the complex drug landscape women navigate [19]. The employment of intravenous drug use in secluded environments, coupled with risky practices such as needle sharing and self-blood injection, underscores the acute health risks WID face. This is consistent with findings by Degenhardt et al. [20], who reported high rates of HIV and HCV transmission among persons who inject drugs due to similar practices. The high-risk practices in this study reflected inconsistent access to commodities, economic constraints, and structural barriers, rather than a total lack of services. The contrast between these findings and the global push towards harm reduction initiatives [21] reveals a gap in targeted interventions for WID in Nigeria, pointing to an urgent need for harm reduction services that are cognizant of local drug use patterns and associated risks.

The preference for bunk locations as the preferred geographical location by WID for accessing health services is a critical finding that reflects on the complex interplay between service accessibility, familiarity, and perceived security. This contrasts with global health service models that prioritize centralized, integrated health services [22], suggesting a nuanced understanding of WID’ preferences and barriers in service utilization. The choice of bunks, underscored by their socio-cultural embeddedness and perceived affordability, necessitates a re-evaluation of current service delivery models for WID, advocating for community-centered approaches that align with their lived realities and preferences. In spite of this preference, bunks can only be for ad-hoc care (e.g., basic first aid, informal supply), akin to outreach, while formal clinical services remain anchored in one-stop shops, pharmacies, and referral facilities. Our findings make a strong case for adopting differentiated service delivery models that adapt to client preferences.

Concerning needles and syringes, the reported ease of access among WID, coupled with their affordability, signifies a critical aspect of harm reduction that is often overlooked in substance use interventions [23]. While this availability is pivotal in reducing the risk of infectious disease transmission, the persistence of high-risk behaviors like needle sharing underscores the complexity of addressing injection drug use practices solely through supply-side strategies. This finding aligns with studies advocating for comprehensive harm reduction services that combine material support with education and behavior change interventions [1], emphasizing the need for multifaceted approaches to effectively mitigate health risks among WID. However, it is important to re-emphasize that reported access to sterile needles in this study largely reflects program-linked distribution (one-stop shops and outreach) and retail purchase in specific locales, rather than universal public provision. Availability and affordability likely remain uneven across states and depend on the presence and activities of implementing partners.

The pervasive issue of GBV among WID, characterized by physical abuse, humiliation, and systemic disenfranchisement, starkly reflects the vulnerabilities they face [4]. The reluctance to report GBV incidents, exacerbated by distrust in law enforcement and fear of retribution, highlights the critical barriers WID encounter in seeking justice and support. This predicament underscores the imperative for integrated GBV and substance use interventions that address the dual spectrums of vulnerability, advocating for protective legal frameworks and accessible support services tailored to the unique needs of WID.

Rehabilitation is a critical element of the care of WID. The deficiency in rehabilitation and follow-up services for WID illuminates a significant gap in the continuum of care necessary for successful recovery and reintegration into society [24]. The absence of gender-sensitive rehabilitation centers and tailored post-rehabilitation support reflect broader systemic failures to address the complex needs of WID, underscoring the urgency for comprehensive rehabilitation services that incorporate gender-specific care and social reintegration support [25].

WID in Nigeria navigate complex socio-economic landscapes, where economic pressures compound the challenges associated with drug use. The combination of economic pressures and legal implications for WID not only perpetuates cycles of drug use but also entrenches their social and economic marginalization [26]. Economic hardship, gender inequality, stigma, and punitive drug policies increase the social and health vulnerabilities of WID, perpetuating their dependency on substances and entrenching their marginalization [27; 28]. The lack of viable economic opportunities, compounded by punitive legal measures, underscores the socio-economic dimensions of substance use disorders among women, echoing the need for policies that transcend criminalization to address the root causes of drug dependency and facilitate economic empowerment and rehabilitation [7].

Findings from this study indicate that the combination of the pressures of poverty, gender inequality, and stringent drug policies heighten the vulnerabilities of WID in Nigeria. Similar structural factors have been identified as major drivers of health risks and barriers to care among WID in sub-Saharan Africa and other low- and middle-income settings [27; 28]. Although evidence among WID in low-resource settings remains limited, prior studies suggest that context-appropriate vocational training and microfinance can lessen reliance on drug-related income and thereby mitigate some attendant risks, [29; 30]. Also, evidence increasingly supports health-centred drug policies, including the decriminalization of personal drug use, as strategies for improving access to prevention, treatment, and harm reduction services while reducing drug-related health harms [27,30]. Taken together, these strands of evidence point to the potential of combining gender-responsive economic empowerment with public-health-oriented drug policy to address immediate health risks while interrupting cycles of poverty and dependence.

The children of WID represent a particularly vulnerable group, often overlooked in interventions designed to address substance use disorders [31,29]. The absence of family-centred services for women who inject drugs may leave their children vulnerable to neglect, social exclusion, and other adverse developmental outcomes, as documented among children affected by parental substance use. Addressing this gap may the development of family-centered interventions that not only focus on the rehabilitation and support of WID but also include educational, nutritional, and psycho-social services for their children [31,29]. Furthermore, community-based initiatives that involve extended family and community members might be able to provide additional layers of support, underscoring the importance of social networks in the recovery process for WID and their families.

Effective client engagement and outreach strategies are foundational for connecting WID with essential services, particularly in contexts where stigma and discrimination inhibit access to care [32,30]. Peer-led outreach and community-based approaches have been widely recommended as effective strategies for improving engagement with women who inject drugs, particularly among populations that experience stigma and limited access to conventional healthcare services [33,30]. Utilizing trusted community members and former WID in outreach roles can significantly enhance programs reach and effectiveness, facilitating access to harm reduction services, healthcare, and social support. Mobile outreach services have been used to extend harm reduction and other health services to populations with limited access to facility-based care. These approaches may complement conventional services by bringing prevention, testing, and referral services closer to women who inject drugs [34,30]. These strategies underscore the importance of flexibility and adaptability in outreach efforts, ensuring that services are accessible to WID in diverse settings.

Advocating for the rights and needs of WID in Nigeria requires careful navigation of cultural sensitivities and societal perceptions that often stigmatize substance use among women [35,30]. Community engagement is an important component of programmes for women who inject drugs because it can improve programme acceptability while reducing stigma and facilitating access to health services [35,30]. Strategies such as community dialogues, engagement with religious and traditional leaders, and media campaigns can help shift perceptions and foster a more supportive environment for WID. Moreover, involving WID in advocacy efforts empowers them as agents of change, providing them with the platform to voice their experiences and contribute to policy development [35,30]. Such participatory approaches are crucial for ensuring that interventions and policies are informed by the lived experiences of WID, enhancing their relevance and effectiveness.

A persistent challenge in addressing the needs of WID in Nigeria is the inadequate funding for targeted interventions. Similar funding gaps have been reported globally, particularly in low- and middle-income countries where harm reduction services rely heavily on donor support [14; 36]. Financial constraints limit the capacity of service providers to offer comprehensive care, including harm reduction services, healthcare, legal aid, and social support. Increasing investment in programs specifically designed for WID is essential for expanding access to services and ensuring their sustainability. Such recommendations have been made in global guidance, which emphasizes the need for dedicated resources to address the unique health and social needs of women who use drugs [14,30]. Strengthening collaboration among government agencies, civil society organizations, development partners, and communities of people who use drugs can play a pivotal role in supplementing local funding, bringing in both financial resources and technical expertise [37,30]. Additionally, integrating WID-focused interventions into broader health and social service frameworks can enhance efficiency and cost-effectiveness, ensuring that WID benefit from existing resources while addressing their specific needs.

### Policy recommendations

Addressing the challenges faced by WID in Nigeria will require a comprehensive approach that prioritizes harm reduction, access to rehabilitation, and supportive legal frameworks. Tailored harm reduction programs may be developed to provide clean needles, counselling, and education on safe injection practices, while integrating services for HIV and Hepatitis B prevention and treatment. Expanding gender-sensitive rehabilitation services may be crucial, incorporating psychological support, vocational training, and social reintegration programs to facilitate recovery. Strengthening legal and policy frameworks may also be necessary to reduce stigma and discrimination against WID, shifting the focus from punitive measures to a health-centered approach that ensures equitable access to services.

Additionally, fostering strong community and family support systems may aid in the reintegration of WID by reducing stigma and providing a stable social environment. Sustainable funding sources may need to be secured to maintain harm reduction and rehabilitation initiatives, leveraging partnerships between government agencies, international organizations, and the private sector. Promoting continuous research and data collection may be essential for understanding the evolving needs of WID and refining intervention strategies. Moreover, involving WID in program development and implementation may help ensure that services are aligned with their lived experiences, potentially increasing engagement and improving outcomes. Implementing these recommendations holistically may lead to a more effective response to the unique challenges faced by WID in Nigeria.

Although heroin, tramadol and pentazocine use/injecting were widely reported, participants and stakeholders did not describe access to methadone, buprenorphine or other medications for opioid use disorder. Treatment narratives centered on counselling/rehabilitation and HIV/hepatitis services, with harm-reduction commodities (needles/syringes, condoms) prominent. This absence may reflect limited medications for opioid use disorder availability/awareness in these settings and warrants dedicated inquiry and programmatic attention.

### Limitation

The research was carried out within the context of ongoing interventions on harm reduction among WID in the study locations. Hence, their perspectives are likely influenced by their experiences with the interventions and may not be representative of other locations where there are no ongoing interventions.

## Conclusion

The discussion surrounding WID in Nigeria reveals a complex interplay of social, economic, and health-related challenges that necessitate a multifaceted and nuanced approach to intervention. The diverse patterns of drug use identified among WID, including the use of both international trafficked and locally concocted substances, underscore the urgent need for targeted harm reduction strategies that are adaptable to the specific contexts and substances involved. Moreover, the preference for accessing health services in informal 'bunk' settings over formal OSS highlights the importance of understanding and integrating the lived experiences and preferences of WID into service delivery. This also makes a strong case for differentiated service delivery. The pervasive issues of gender-based violence, economic instability, and the stigmatization faced by WID further compound their vulnerability, indicating the critical need for comprehensive interventions that address both the immediate health risks associated with injection drug use and the broader socio-economic factors at play. The study has identified gaps in specific training for handling WID, inclusion of their children in programs, and follow-up during rehabilitation.

Efforts to improve the situation for WID in Nigeria must therefore prioritize the development and implementation of gender-sensitive, culturally appropriate harm reduction services that acknowledge the complexity of WID experiences. The role of community engagement, family support, and the legal context in shaping WID’s access to and utilization of services cannot be overstated, highlighting the need for collaborative approaches that bring together government, non-governmental organizations, healthcare providers, and the WID themselves. Funding for such interventions, alongside advocacy for policy reforms aimed at decriminalizing drug use and reducing the stigma and discrimination faced by WID, is essential for creating a more supportive environment for WID recovery and integration into society. Ultimately, addressing the needs of WID in Nigeria requires a commitment to understanding their unique challenges and working collaboratively across sectors to provide holistic, effective support.

## Supporting information

S1 Data Barriers to Harm Reduction Services among WID. (XLSX)
