# Supplementary material for: Understanding barriers to healthcare and harm reduction services among women who inject drugs in Nigeria: A qualitative assessment
Source: PLOS Glob Public Health. 2026 Aug 13;6(8):e0007008. doi: 10.1371/journal.pgph.0007008 (PMC13472433; doi:10.1371/journal.pgph.0007008)
Supplement: Supplementary file 1 [file pgph.0007008.s002.docx]

Thank you for the opportunity to read this important manuscript. This is a qualitative study focused on understanding the current state of health care access and utilization among women who inject drugs in Nigeria, using key informant interviews of stakeholders and focus group discussions of women who inject drugs. This is an important topic, given the lack of information in the current literature on women who inject drugs in sub-Saharan Africa and the need for tailored services to women who have unique needs compared to men who use drugs. Some additional background in the introduction and discussion would provide greater context for an international audience, and the methods could be expanded upon for clarity.

Introduction:

- I would provide more background in the introduction on the current landscape of drug use and injection drug use in Nigeria (opioids, stimulants, etc), in the general population and among women. How common is this, and what are the most used drugs in Nigeria (or sub-Saharan Africa)? What is the current landscape of substance use treatment? For example, if opioids are common, is opioid agonist treatment (“medication for opioid use disorder” or “medication assisted treatment”) like methadone or buprenorphine available? Are syringe service programs or needle exchange programs legal? Since this manuscript is focused on women, what are the local laws or policies around substance use during pregnancy? Are they punitive? Understanding this local context is important for interpreting the results/findings of this study.

Methods:

- Some key elements are missing in the methods. I recommend referring to COREQ guidelines as a checklist for comprehensive reporting for qualitative studies. Specific examples include:
  - How were the women who inject drugs recruited for the focus group discussions? Supposedly 60 women (6 focus group discussions of 10 women each) were recruited within 3 weeks, according to the methods. This is impressive for a qualitative study, and I am curious how this was accomplished and if it was a representative sample of the region, or all from one clinic?
  - How were these women contacted or reached? What was the sampling strategy? How many were contacted vs agreed to participate?
  - Inclusion/exclusion criteria?
  - Who conducted the interviews? For example, what were their credentials, gender, and training? This is relevant because these participants are being interviewed about a topic that is stigmatized, so their responses may differ if being interviewed by all male interviewers or if there is a component of hierarchy. Did the people conducting the interviews have a relationship with the places where WID were recruited? (I noticed on page 11 that one respondent said “but you people do not charge us”—does that mean the interviewers worked at the facility where the respondent was recruited from?)
  - Were participants compensated?
  - What language were the interviews conducted in? Are different languages spoken in the different regions, and if so, how was this handled?
  - How were data recorded or transcribed? What (if any) software or tools were used to transcribe the interviews?
- Were there any clinical sites or clinicians interviewed as part of this study, and if not, why not? It seems that it would have been helpful to have the perspective of health care workers as well.

Results:

- Basic demographics: Were basic demographic info collected? I would like to have seen a paragraph or table under the characteristics of women who inject drugs that shows the basic demographics of the respondents, particularly the women who inject drugs. Do we know their ages? If HIV status, pregnancy status, marital status were available, those would be pertinent information. Marital status is relevant because of the inclusion of topic of gender based violence in the paper.
- Characteristics of the stakeholders would have been nice to see as well, if available, as it would inform any potential biases in the responses.
- For Methods of Injection section, did we ask where they typically get their needles? (Pharmacy, friends, or needle exchange programs?)
- While reading the results, I could not tell if the WID were all recruited from the OSS clinic and giving feedback on that single program, or describing substance use care in general across Nigeria. It seemed like they were giving feedback about a specific program, and I was surprised to see the generally positive feedback about the substance use care. This is where clarifying the recruitment strategy in the Methods would be helpful. If all the participants were recruited from the same clinic and were interviewed by staff associated with the clinic, responses may be biased.
- On page 9, authors report that respondents from Abia and Oyo seem to be better informed on the matter (of safe injection practices). This is author interpretation, better suited for the discussion, not the results.
- Did the qualitative interviews ask about access to perinatal/antenatal care for WID?

Discussion:

- I was confused about whether the bunk location was where participants wished care was delivered, or if care was currently already being delivered there. Are there health care delivery programs at the bunks?
- I was surprised by the ease of access of clean needles that was reported. Again, it would be helpful to know the current landscape of needle exchange programs in Nigeria.
- I found the inclusion of gender-based violence as part of this qualitative study to be very insightful and profoundly important! Thanks for including this.
- Since opioid use was reported, I wished they had asked more about access to medications for opioid use disorder (e.g. methadone, buprenorphine) since this is the gold standard in many countries for treating opioid use disorder, rather than rehabilitation alone.
- Some of the recommendations proposed in the discussion were not aligned with the study’s own findings. For example, authors mention providing microfinance opportunities to reduce reliance on drug related income, or to cater to the needs of children. I agree with both, however I did not remember seeing these as barriers to care reported in the results section.

Other minor points:

- Several areas of duplication in spelling out women who inject drugs (WID). After defining this acronym in the paper, no need to spell out again.
- I would reduce acronyms where able. If not used frequently in the manuscript, I would just spell out the term and not use the acronym, as it’s difficult to follow for new readers. For example, a lot of acronyms on pages 4-6.
- Page 8 – “WID access healthcare from a range of venues” Is reported in the results. Could authors be more specific about the types of venues where WID access care, in addition to just the bunk?
