# Supplementary material for: Understanding barriers to healthcare and harm reduction services among women who inject drugs in Nigeria: A qualitative assessment
Source: PLOS Glob Public Health. 2026 Aug 13;6(8):e0007008. doi: 10.1371/journal.pgph.0007008 (PMC13472433; doi:10.1371/journal.pgph.0007008)
Supplement: Supplementary file 2 [file pgph.0007008.s003.docx]

**Point-by-point response to reviewers**

**General**

**Reviewer summary:**

Thank you for the opportunity to read this important manuscript. This is a qualitative study focused on understanding the current state of health care access and utilization among women who inject drugs in Nigeria, using key informant interviews of stakeholders and focus group discussions of women who inject drugs. This is an important topic, given the lack of information in the current literature on women who inject drugs in sub-Saharan Africa and the need for tailored services to women who have unique needs compared to men who use drugs. Some additional background in the introduction and discussion would provide greater context for an international audience, and the methods could be expanded upon for clarity.

**Response:** We appreciate the detailed review and agree with the requested clarifications and expansions. We have revised the manuscript to add country background, strengthen methods per COREQ, and sharpen the results–discussion linkage. We have inserted new subsections, tables, and cross-references where indicated below. Where data were not collected, we now state this explicitly and explain the impact on interpretation. All line edits are tracked in the revised manuscript and detailed below by section.

**Comment**

- I would provide more background in the introduction on the current landscape of drug use and injection drug use in Nigeria (opioids, stimulants, etc), in the general population and among women. How common is this, and what are the most used drugs in Nigeria (or sub-Saharan Africa)? What is the current landscape of substance use treatment? For example, if opioids are common, is opioid agonist treatment (medication for opioid use disorder or medication assisted treatment) like methadone or buprenorphine available? Are syringe service programs or needle exchange programs legal? Since this manuscript is focused on women, what are the local laws or policies around substance use during pregnancy? Are they punitive? Understanding this local context is important for interpreting the results/findings of this study.

**Response:** We have inserted the following information:

*Among WID in Nigeria, injected substances reported in local sources include pentazocine, tramadol (including melted formulations), heroin, and cocaine (UNODC, 2018). Opioid agonist treatment (methadone, buprenorphine) remains limited relative to need, and needle/syringe provision occurs mainly through implementing-partner platforms (e.g., one-stop shops and outreach) rather than universal public coverage (Harm Reduction International, 2022; UNODC, 2018).*

**Comment**

- Some key elements are missing in the methods. I recommend referring to COREQ guidelines as a checklist for comprehensive reporting for qualitative studies. Specific examples include:
o How were the women who inject drugs recruited for the focus group discussions? Supposedly 60 women (6 focus group discussions of 10 women each) were recruited within 3 weeks, according to the methods. This is impressive for a qualitative study, and I am curious how this was accomplished and if it was a representative sample of the region, or all from one clinic?

o How were these women contacted or reached? What was the sampling strategy? How many were contacted vs agreed to participate?

o Inclusion/exclusion criteria?

o Who conducted the interviews? For example, what were their credentials, gender, and training? This is relevant because these participants are being interviewed about a topic that is stigmatized, so their responses may differ if being interviewed by all male interviewers or if there is a component of hierarchy. Did the people conducting the interviews have a relationship with the places where WID were recruited? (I noticed on page 11 that one respondent said but you people do not charge us—does that mean the interviewers worked at the facility where the respondent was recruited from?)

o Were participants compensated?

o What language were the interviews conducted in? Are different languages spoken in the different regions, and if so, how was this handled?

o How were data recorded or transcribed? What (if any) software or tools were used to transcribe the interviews?

Were there any clinical sites or clinicians interviewed as part of this study, and if not, why not? It seems that it would have been helpful to have the perspective of health care workers as well.

**Response:** We have restructured Methods with COREQ headings and added the missing elements. The statement *‘but you people do not charge us’* refers to the outreach workers/programme, not the research team. Below is the revised text:

*The study's participants were drawn from a diverse group of stakeholders involved in drug use management and health service provision for WID. Key informant interviews targeted agencies interfacing routinely with WID: Harm Reduction Technical Working Group, State Agency for the Control of AIDS (SACA), Police Action Committee on AIDS (PACA), National Drug Law Enforcement Agency (NDLEA), Key Population Secretariat, Association of Women Living with HIV/AIDS in Nigeria (ASHWAN), Legal Aid Council, and the National Human Rights Commission (NHRC). However, no clinicians were interviewed. This design prioritised lived experience and programme/structural views aligned with study objectives.*

*We used purposive sampling to recruit 60 WID across urban and rural communities for the focus group discussions (FGD). Recruitment took place directly at hotspots where WID congregate some of whom are part of ongoing interventions. An outreach worker who routinely engages with WID at the hotspots approached potential participants face-to-face, described the study, and obtained initial agreement. Phone contacts were collected, and reminders were provided one day before each session. Over 20 women were initially contacted per site; those available on scheduled dates were transported to the venues for the FGD session. Incision criteria were Inclusion: female; age 18–49 years; currently injects drugs; physically able to participate; provides verbal informed consent; agrees to audio recording. Exclusion: age <18 or >49; does not currently inject; not resident in the study state; unable to participate physically; declines consent or audio recording. Six FGDs were conducted (2 per state, 10 participants per group). Recruitment and enrolment were completed within three weeks, enabled by close social networks and peer referral. The sample is not statistically representative of the region; it reflects WID within the selected bunks.*

*FGDs and in-depth interviews were conducted by female research assistants contracted for the study. All held at least a university degree and prior qualitative experience. A one-day refresher covered research ethics, interviewing techniques, and handling sensitive topics. Interviewers had no prior relationships with participants and were independent of recruitment locations. Outreach workers (implementing organisation staff) handled mobilisation and transport only; they did not conduct interviews.*

*Table 1 below shows the different categories of study participants. Also, Box 1 below briefly describes the agencies/organization/group from which the stakeholders were drawn from. No financial incentives were provided. Participants received refreshments during sessions and transport money for return travel (for those who incurred transportation cost), per ethics approvals.*

**Results**

**Comment:**

**-** Basic demographics: Were basic demographic info collected? I would like to have seen a paragraph or table under the characteristics of women who inject drugs that shows the basic demographics of the respondents, particularly the women who inject drugs. Do we know their ages? If HIV status, pregnancy status, marital status were available, those would be pertinent information. Marital status is relevant because of the inclusion of topic of gender-based violence in the paper.

- Characteristics of the stakeholders would have been nice to see as well, if available, as it would inform any potential biases in the responses.

**Response:** We have inserted the following

*Detailed demographics were intentionally not collected to reduce re-identification risk while program records verified adult status (18–49 years), non-pregnancy, and HIV-negative status for all FGD participants. For key informants, we recorded institutional role and organizational affiliation only, as our focus was on role-specific perspectives rather than personal characteristics.*

**Comment**

- For Methods of Injection section, did we ask where they typically get their needles? (Pharmacy, friends, or needle exchange programs?)

**Response:** There is a subsection titled “Needles and syringes” that addresses their source. We made the following up in the said section

*Participants described consistently available sterile injecting equipment delivered through one-stop shops and community outreach, with additional access at bunk-based activities and, at times, through retail medicine stores/patent and proprietary medicine vendors (PPMV). Distribution was portrayed as routine and oriented toward single-use to reduce sharing, and formal needle–syringe exchange/return was linked to OSS operations and reported during some bunk-side engagements. A smaller group still relied on peers’ equipment when supplies were unavailable or unaffordable. The following quotes for study participants.*

**Comment**

- While reading the results, I could not tell if the WID were all recruited from the OSS clinic and giving feedback on that single program, or describing substance use care in general across Nigeria. It seemed like they were giving feedback about a specific program, and I was surprised to see the generally positive feedback about the substance use care. This is where clarifying the recruitment strategy in the Methods would be helpful. If all the participants were recruited from the same clinic and were interviewed by staff associated with the clinic, responses may be biased.

**Response:** FGD participants were recruited via multiple entry points (bunks, hotspots, peer referral) and were interviewed by independent research staff who were not part of service delivery. Reported experiences therefore reflect both programme-linked and community settings rather than a single clinic. We have provided this information in the methodology

**Comment**

- On page 9, authors report that respondents from Abia and Oyo seem to be better informed on the matter (of safe injection practices). This is author interpretation, better suited for the discussion, not the results.

**Response:** We have deleted this discussion level material from the Results

**Comment**

- Did the qualitative interviews ask about access to perinatal/antenatal care for WID?

**Response:** We have provided the following revision

*Access to antenatal care (ANC) was discussed across Abia, Oyo, and Gombe: several participants described prior ANC use and prevention-of-mother-to-child-transmission (PMTCT) support—“It’s antenatal… Yes, I have accessed it before” (FGD participant/Abia); “it is possible you have HIV and you are pregnant so they will look for a way to be giving you drugs so that the child will not be affected… and after delivery they also make sure that they monitor you” (FGD participant /Gombe). At the same time, others linked inconsistent ANC to stigma and fear of disclosure—“when they are pregnant… due to that stigmatization, they don't go to hospital… They start to… shade themselves away from the public” (FGD participant /Abia); some “don’t go to antenatal” at all (FGD participant/Abia), and negative facility encounters reinforced avoidance—“the way they treated her that day… anytime I have problem… I don't bother to go” (FGD participant /Abia). In Oyo, women described concealing drug use when seeking care—“I wouldn’t let anyone know I’m a WID at all… because of the discrimination and stigmatization” (FGD participant /Oyo)—underscoring how stigma undermines consistent ANC uptake despite reported availability.*

**Discussion**

**Comment**

- I was confused about whether the bunk location was where participants wished care was delivered, or if care was currently already being delivered there. Are there health care delivery programs at the bunks?

**Response:** We have provided the following revision

*In spite of this preference, bunks can only be for ad-hoc care (e.g., basic first aid, informal supply), akin to outreach, while formal clinical services remain anchored in one-stop shops, pharmacies, and referral facilities.*

**Comment**

- I was surprised by the ease of access of clean needles that was reported. Again, it would be helpful to know the current landscape of needle exchange programs in Nigeria.

**Response:** We have provided the following revision

*However, it is important to re-emphasise that reported access to sterile needles in this study largely reflects programme-linked distribution (one-stop shops and outreach) and retail purchase in specific locales, rather than universal public provision. Availability and affordability remain uneven across states and depend on the presence and activities of implementing partners*.

**Comment**

- I found the inclusion of gender-based violence as part of this qualitative study to be very insightful and profoundly important! Thanks for including this.

**Response:** Appreciated. No text change required.

**Comment**

- Since opioid use was reported, I wished they had asked more about access to medications for opioid use disorder (e.g. methadone, buprenorphine) since this is the gold standard in many countries for treating opioid use disorder, rather than rehabilitation alone.

**Response:** When treatment was discussed, it was framed as counselling/rehab or HIV services, and where “drugs” were referenced they were unspecified or HIV-related (e.g., PrEP), not MOUD. This suggests either limited availability and/or low awareness of MOUD in the study settings, and we agree this is an important gap to note in the limitations and recommendations.

**Comment**

- Some of the recommendations proposed in the discussion were not aligned with the study’s own findings. For example, authors mention providing microfinance opportunities to reduce reliance on drug related income, or to cater to the needs of children. I agree with both, however I did not remember seeing these as barriers to care reported in the results section.

**Response:** We revised the indicated recommendations as follows:

*Findings from this study indicate that the combination of the pressures of poverty, gender inequality, and stringent drug policies heighten the vulnerabilities of women who inject drugs (WIDs) in Nigeria, echoing broader evidence from Sub-Saharan Africa that calls for integrated responses (Adelekan et al., 2021). Prior studies suggest that context-appropriate vocational training and microfinance can lessen reliance on drug-related income and thereby mitigate some attendant risks (Otiashvili et al., 2018). Also, legal reforms that decriminalize personal drug use and prioritize health-centred responses are associated with reduced harms, particularly for women (West et al., 2020). Taken together, these strands of evidence point to the potential of combining gender-responsive economic empowerment with public-health-oriented drug policy to address immediate health risks while interrupting cycles of poverty and dependence.*

**Other minor points**

**Comment**

- Several areas of duplication in spelling out women who inject drugs (WID). After defining this acronym in the paper, no need to spell out again.

**Response:** Global edit: after first definition, use ‘WID’ only, except in direct quotations or titles.

**Comment**

- I would reduce acronyms where able. If not used frequently in the manuscript, I would just spell out the term and not use the acronym, as it’s difficult to follow for new readers. For example, a lot of acronyms on pages 4-6.

**Response**: Global edit: remove low-frequency acronyms; retain only those essential to programme context with a concise abbreviations list.

**Comment**

- Page 8 – WID access healthcare from a range of venues Is reported in the results. Could authors be more specific about the types of venues where WID access care, in addition to just the bunk?

**Response:** We have updated the information to indicate the women accessed services via one-stop shops, pharmacies, public hospitals/clinics, mobile/outreach points, and bunk settings, with choices shaped by perceived safety, cost, and travel distance.
