# Supplementary material for: Understanding barriers to healthcare and harm reduction services among women who inject drugs in Nigeria: A qualitative assessment
Source: PLOS Glob Public Health. 2026 Aug 13;6(8):e0007008. doi: 10.1371/journal.pgph.0007008 (PMC13472433; doi:10.1371/journal.pgph.0007008)
Supplement: Supplementary file 3 [file pgph.0007008.s004.docx]

**Response to reviewers**

**Reviewer #1**

**Comment:**
Thank you for the opportunity to review your revised manuscript. This is a qualitative study of women who inject drugs in Nigeria exploring the current landscape of health care access and utilization. Strengths noted include the importance of the topic, strong qualitative approach, inclusion of both women who inject drugs and stakeholders, and a large qualitative sample size. The reviewer stated that all comments were adequately addressed and recommended acceptance.

**Response:** We thank the reviewer for the careful reading of our manuscript and for the positive assessment of the study’s relevance, qualitative design, and analytic breadth. We appreciate the recognition of the inclusion of both women who inject drugs and key stakeholders, which strengthened the study’s ability to capture lived experience alongside programmatic and structural perspectives. We are grateful for the recommendation for acceptance.

**Reviewer #2**

**Main comments**

**Comment 1**

There could be some tidying up of the methods to clarify the process for interviews and focus group discussions. I suggest separating these two in the Data Collection subsection and providing rationale for this approach.

**Response:** We have separated key informant interviews and focus group discussions within the Data Collection section and have added the rationale for using both methods. KIIs were used to capture service, policy, legal, and program perspectives, while FGDs were used to capture shared lived experiences, service preferences, and barriers among women who inject drugs.

**Comment 2**

It is not fully clear how the themes emerged from the data and what was pre-determined by the interviewer. The aims also read too quantitatively.

**Response:** We agree. We have revised the study aim to reflect an exploratory qualitative purpose. We have also clarified that the interview guides covered broad domains relevant to service access and safety, while gender-based violence emerged strongly as a cross-cutting analytic theme during coding and interpretation.

**Comment 3**

There are spelling and grammatical errors. A full proof-read is needed.

**Response:** We conducted a language edit across the manuscript including correction of spelling, grammar, punctuation, subject-verb agreement, repeated words, incomplete quotations, inconsistent capitalization, and formatting problems.

**Comment 4**

A table showing services, whether they are available, and where they are available would be useful.

**Response:** We agree. We have added a summary table to present reported service availability in a more accessible format. We clarify in the table title that this is a qualitative summary of reported availability rather than a formal service inventory.

**Comment 5**

The perspective of participants is often unclear. It is not always obvious whether the statements come from women with lived experience or from service providers.

**Response:** We have revised the Results section throughout to identify the source of each finding more clearly. We now distinguish statements from FGDs, KIIs, and where relevant, both. FGDs were only carried out among the women, representing their lived experiences, while KII were only with service providers

**Abstract**

**Comment 1**

Suggest not using acronym WID and using acronyms sparingly.

**Response:** We have removed the acronym from the title and abstract and reduced acronym use across the manuscript. We retain abbreviations only where repeated use makes the text easier to read. Also, earlier reviewer had drawn out attention to the need to use acronyms after full mean have been stated at the first use. We have tried to keep to this suggestion

**Comment 2**

The title says harm reduction access and the Introduction says healthcare access. Use consistent terminology.

**Response:** We have harmonized the terminology across the manuscript by using “healthcare and harm reduction services” where the broader scope is intended. We have also revised the title to reflect this broader scope.

**Comment 3**

Error “ATLAS.ti 24” in last line of Methodology section.

**Response:** The sentence has been corrected. We have also completely revised the abstract

**Introduction**

**Comment 4**

The statement on blood-borne viruses reads as if these risks are unique to women.

**Response:** We have revised the sentence to avoid implying that these infections are unique to women and instead focus on gendered vulnerability and unmet service needs.

**Comment 5**

The Degenhardt (2013) paper does not support the violence statement.

**Response:** We have removed that citation from the violence statement and replaced it with references that are more directly relevant to violence among women who use drugs.

**Comment 6**

References are needed in paragraph 3.

**Response:** We have added references to support statements on stigma, exclusion, and lack of gender-specific support.

**Comment 7**

Typo: “and many face significant barriers”.

**Response:** Corrected.

**Comment 8**

The aims use language that sounds quantitative.

**Response:** We agree and have revised the aim accordingly.

**Methodology**

**Comment 9**

Typo in section 2.3: “Incision criteria were Inclusion”.

**Response:** Corrected to “Inclusion criteria were”.

**Comment 10**

IDI in the table is not defined, and the table title is missing.

**Response**: We conducted key informant interviews (KII) and not in-depth interviews (IDI), We have corrected the tables accordingly

**Comment 11**

Please clarify the number of focus groups and stakeholders.

**Response:** We have clarified this directly in the text. The study included six FGDs with women who inject drugs, with 10 participants in each group, and 24 KIIs across eight stakeholder categories.

**Comment 12**

The box could be moved higher under the first paragraph of Study population and Sampling.

**Response:** We agree and have moved Box 1 to improve flow.

**Comment 13**

Who was asked the semi-structured interviews?

**Response:** We have clarified that the interviews were the KIIs conducted with stakeholder participants, while the FGDs were conducted with women who inject drugs.

**Comment 14**

Suggest a separate sub-heading called Data analysis.

**Response:** We have separated Data Analysis from Data Collection.

**Comment 15**

Worth noting when participants were de-identified.

**Response:** We agree and have added this detail.

**Results**

**Comment 16**

Please clarify whether pregnancy and HIV positivity were exclusion criteria.

**Response:** We agree that the previous wording could be misread. We have revised the text to avoid implying additional exclusion criteria from the Results section.

**Comment 17**

I question whether “typologies” is the correct term.

**Response:** We have replaced “typologies” with a more accurate phrase.

**Comment 18**

Are these typologies mutually exclusive?

**Response:** The ‘typologies’ are not mutually exclusive. The revised paragraph now states that these are overlapping descriptive groupings rather than mutually exclusive categories.

**Comment 19**

The perspective is difficult to follow in some places.

**Response:** We revised the text throughout the Results to specify whether the finding came from stakeholders, women in FGDs, or both.

**Comment 20**

There is a repeat quote at the end of page 10.

**Response:** The duplicate quote has been removed.

**Comment 21**

The acronym OSS is inconsistently used.

**Response:** We have now defined “one-stop shop (OSS)” at first mention and use “OSS” consistently thereafter.

**Comment 22**

Inconsistent use of quotation marks around participant quotes.

**Response:** We standardized quotation formatting throughout according to one style.

**Comment 23**

“Antenatal care services are also accessible” appears in italics but seems to be narrative text.

**Response:** Corrected. The sentence is now presented as standard narrative text.

**Comment 24**

Instead of “XXX”, use bracketed descriptors.

**Response:** We have replaced placeholders such as “XXX” with bracketed descriptors, for example “[hospital name]” or “[implementing organisation]”, where needed.
