# Supplementary material for: Understanding barriers to healthcare and harm reduction services among women who inject drugs in Nigeria: A qualitative assessment
Source: PLOS Glob Public Health. 2026 Aug 13;6(8):e0007008. doi: 10.1371/journal.pgph.0007008 (PMC13472433; doi:10.1371/journal.pgph.0007008)
Supplement: Supplementary file 4 [file pgph.0007008.s005.docx]

**Response to reviewers**

We thank the reviewers for their additional detailed feedback. We have completed a full revision focusing on clarity, reduction and standardization of abbreviations, correction of all language errors, and strengthening of the abstract and conclusion. We also refined the methodological description, clarified thematic development, and aligned the discussion and conclusion more closely with participant-derived findings

**Reviewer #1**

**General comment**

**Comment:**
The manuscript is stronger, but there are still misspellings and misuse of abbreviations.

**Response:**
We agree. We conducted a full proof-read and standardization of abbreviations. Abbreviations are now limited to those used repeatedly. All spelling, grammar, and formatting errors have been corrected.

**Abstract**

1. **Comment 1**

STI, PMTCT, and WID need to be defined.

**Response:**
We agree. All acronyms are now defined at first use in the abstract. We have completely re-written the abstract

and confidential referral systems that reduce exposure to stigma and law enforcement risk.

2. **Comment 2**

“Availability varied…” is vague.

**Response:**
We replaced this with specific patterns

3. **Comment 3**

“Bunks” not universally known.

**Response:**
We retained the term and added quotation marks to indicate contextual meaning in the abstract.

4. **Comment 4**

Conclusion is vague.

**Response:**
We replaced general statements with specific programmatic actions.

**Introduction**

5. **Comment 5**

Replace “drug users” with person-first language.

**Response:**
Corrected throughout text.

6. **Comment 6**

Replace “drug abuse” with prescription drug misuse.

**Response:**
Corrected throughout.

7. **Comment 7**

Avoid unnecessary abbreviations.

**Response:**
We removed low-frequency abbreviations (e.g., IPV).

8. **Comment 8**

Use consistent terminology for treatment.

**Response:**
We replaced instances with “medications for opioid use disorder” where relevant

9. **Comment 9**

PWID defined twice.

**Response:**
Duplicate definition removed.

**Methods**

10. **Comment 10**

Too many abbreviations.

**Response:**
We reduced abbreviations to: KII, OSS, NSP, WID, and FGD, as well as names of organizations with formally recognized abbreviations. All others removed or written in full.

11. **Comment 11**

FGD defined multiple times.

**Response:**
Now defined once at first use only.

12. **Comment 12**

Spelling error “enrolment”.

**Response:**
Corrected.

13. **Comment 13**

Were participants compensated?

**Response:**
We added clarification.

“Participants did not receive financial compensation. Transportation and refreshments were provided to cover attendance costs.”

14. **Comment 14**

Typo in Data Collection opening sentences.

**Response:**
Corrected and simplified.

“Participant recruitment occurred between October 9-31, 2023 while data collection was conducted from November 13 to 30, 2023.”

15. **Comment 15**

Was saturation reached?

**Response:**
We added explicit statement.

“Data collection was driven by maximum variation sampling to achieve thematic saturation”

**Results**

16. **Comment 16**

Section 3.1.1 belongs in Methods.

**Response:**
We moved this section to Methods under “Study population and sampling.”

17. **Comment 17**

Inconsistent use of WID.

**Response:**
We standardized usage across the manuscript. “WID” is now used consistently after first definition, except at sentence starts.

18. **Comment 18**

Typo “interveiwed”.

**Response:**
Corrected.

19. **Comment 19**

Table 2 appreciated.

**Response:**
No change required.

20. **Comment 20**

Rename section 3.6.

**Response:**
We revised the title (now section 3.5).

**Revised heading:**
**3.5 Potential facilitators to improving healthcare access for women who inject drugs**

**Discussion**

21. **Comment 21**

Link to differentiated service delivery.

**Response:**
We incorporated this concept.

“Our findings make a strong case for adopting differentiated service delivery models that adapt to client preferences.”

22. **Comment 22**

Why does risky behavior persist?

**Response:**
We clarified without speculation beyond data.

**“**The high-risk practices in this study reflected inconsistent access to commodities, economic constraints, and structural barriers, rather than a total lack of services.”

23. **Comment 23**

Typo “on-gong”.

**Response:**
Corrected to “ongoing”.

**Conclusion**

24. **Comment 24**

“International narcotics” unclear.

**Response:**
“…including the use of both international trafficked and locally concocted substances”.

25. **Comment 25**

State differentiated service delivery clearly.

**Response:**
Added.

26. **Comment 26**

Rehabilitation centers overemphasized.

**Response:**
We agree and revised the conclusion accordingly.

**Reviewer #2**

27. **Comment 1**

Remove second sentence in Data Collection.

**Response:**
Removed as suggested.

28. **Comment 2**

Include definitions for acronyms in table notes.

**Response:**
We added table notes.

“Note: WID = women who inject drugs; FGD = focus group discussion; STI = sexually transmitted infection; PMTCT = prevention of mother-to-child transmission; NHRC = National Human Rights Commission; OSS = one-stop shop.”

29. **Comment 3**

Acronyms still overused.

**Response:**
We further reduced acronym use across the manuscript. Only high-frequency terms are retained. All single-use acronyms have been removed.

We thank the reviewers for their additional detailed feedback. We have completed a full revision focusing on clarity, reduction and standardization of abbreviations, correction of all language errors, and strengthening of the abstract and conclusion. We also refined the methodological description, clarified thematic development, and aligned the discussion and conclusion more closely with participant-derived findings
