# Supplementary material for: Understanding barriers to healthcare and harm reduction services among women who inject drugs in Nigeria: A qualitative assessment
Source: PLOS Glob Public Health. 2026 Aug 13;6(8):e0007008. doi: 10.1371/journal.pgph.0007008 (PMC13472433; doi:10.1371/journal.pgph.0007008)
Supplement: Supplementary file 5 [file pgph.0007008.s006.docx]

**Response to reviewers**

i) Please address Reviewer 1's comment about data saturation. The statement currently in the manuscript does not provide any details on how saturation was assessed or whether it was reached.

**Response**

We thank the reviewer for this important methodological query. On reflection, we recognise that "data saturation" in the strict empirical sense — as originally conceptualised by Glaser and Strauss — refers to the point at which no new themes or codes emerge during concurrent data collection and analysis. Our approach did not involve this iterative saturation assessment during fieldwork. Instead, sample adequacy was determined a priori using a predetermined data sources and maximum variation sampling strategy: before data collection, we specified the number of states, the number of focus group discussions per state, and the key stakeholder groups relevant to women who inject drugs, in order to ensure that the sampling frame would capture the anticipated range of variation in experiences and perspectives across the three states.

We have revised the manuscript to reflect this more precisely, replacing references to "data saturation" with "sampling adequacy" / "information power," consistent with Malterud et al.'s (2016) framework, which argues that sample size and coverage can be justified a priori based on study aims, sample specificity, and analytical strategy, rather than solely through post hoc saturation assessment. We believe this revised framing more accurately represents our methodological approach and addresses the reviewer's concern. The relevant text now reads as follows:

Rather than determining saturation through iterative assessment during data collection, sample adequacy in this study was informed by a predetermined data sources and maximum variation strategy. Before data collection, we specified the number of states, the number of FGDs per state, and the key stakeholder groups relevant to women who inject drugs, so that the sampling frame would capture the anticipated range of variation in experiences and perspectives across settings. This a priori approach — sometimes referred to as an information power or sampling adequacy strategy — was designed to ensure adequate coverage of relevant sites and stakeholder groups rather than to confirm saturation empirically through concurrent analysis (Malterud et al. 2016).

ii) Please revisit the Reviewer's comments about the section in the Conclusions regarding rehabilitation centers. As the Reviewer notes, this is not reflected in WID interviews. I suggest a more nuanced discussion of this point reflecting the difference in the views of stakeholders vs WID.

**Response**

We have completely removed the portion of the statement calling for urgent provision of rehabilitation centers since this is not reflected in WID interviews
