# Supplementary material for: Understanding barriers to healthcare and harm reduction services among women who inject drugs in Nigeria: A qualitative assessment
Source: PLOS Glob Public Health. 2026 Aug 13;6(8):e0007008. doi: 10.1371/journal.pgph.0007008 (PMC13472433; doi:10.1371/journal.pgph.0007008)
Supplement: Peer review history

# Peer review: Decision Letter 0

**DOI:** 10.1371/journal.pgph.0007008.r001

**Authors:** Katherine Demi Kokkinias

26 Aug 2025

PGPH-D-25-01672

Understanding Barriers to Harm Reduction Services Among Women who Inject Drugs in Nigeria: a qualitative assessment

PLOS Global Public Health

Dear Dr. Arije,

Thank you for submitting your manuscript to PLOS Global Public Health. After careful consideration, we feel that it has merit but does not fully meet PLOS Global Public Health’s publication criteria as it currently stands. Therefore, we invite you to submit a revised version of the manuscript that addresses the points raised during the review process.

==============================

Please note that we have only been able to secure a single reviewer to assess your manuscript, their comments are available below. We are issuing a decision on your manuscript at this point to prevent further delays in the evaluation of your manuscript. Please be aware that the editor who handles your revised manuscript might find it necessary to invite additional reviewers to assess this work once the revised manuscript is submitted. However, we will aim to proceed on the basis of this single review if possible.

The reviewer raised a number of major concerns. They feel the manuscript would benefit from additional background information in the introduction and discussion, and they request improvements to the reporting of methodological aspects of the study, for example, regarding the inclusion/exclusion criteria and more information describing the interviews. The reviewers also raised questions about basic demographics information and knowledge of respondents.

Could you please carefully revise the manuscript to address all comments raised?

==============================

Please submit your revised manuscript by Oct 03 2025 11:59PM. If you will need more time than this to complete your revisions, please reply to this message or contact the journal office at globalpubhealth@plos.org. When you're ready to submit your revision, log on to https://www.editorialmanager.com/pgph/ and select the 'Submissions Needing Revision' folder to locate your manuscript file.

Please include the following items when submitting your revised manuscript:

- A rebuttal letter that responds to each point raised by the editor and reviewer(s). You should upload this letter as a separate file labeled 'Response to Reviewers'.
- A marked-up copy of your manuscript that highlights changes made to the original version. You should upload this as a separate file labeled 'Revised Manuscript with Track Changes'.
- An unmarked version of your revised paper without tracked changes. You should upload this as a separate file labeled 'Manuscript'.

Guidelines for resubmitting your figure files are available below the reviewer comments at the end of this letter.

We look forward to receiving your revised manuscript.

Kind regards,

Katherine Demi Kokkinias, Ph.D.

Staff Editor

PLOS Global Public Health

Journal Requirements:

If the reviewer comments include a recommendation to cite specific previously published works, please review and evaluate these publications to determine whether they are relevant and should be cited. There is no requirement to cite these works unless the editor has indicated otherwise.

Additional Editor Comments (if provided):

[Note: HTML markup is below. Please do not edit.]

Reviewers' comments:

Reviewer's Responses to Questions

**Comments to the Author**

1. Does this manuscript meet PLOS Global Public Health’s publication criteria?

Reviewer #1: Partly

**********

2. Has the statistical analysis been performed appropriately and rigorously?

Reviewer #1: N/A

**********

3. Have the authors made all data underlying the findings in their manuscript fully available (please refer to the Data Availability Statement at the start of the manuscript PDF file)?

The PLOS Data policy

Reviewer #1: Yes

**********

4. Is the manuscript presented in an intelligible fashion and written in standard English?

Reviewer #1: Yes

**********

Reviewer #1: Thank you for the opportunity to read this important manuscript. This is a qualitative study focused on understanding the current state of health care access and utilization among women who inject drugs in Nigeria, using key informant interviews of stakeholders and focus group discussions of women who inject drugs. This is an important topic, given the lack of information in the current literature on women who inject drugs in sub-Saharan Africa and the need for tailored services to women who have unique needs compared to men who use drugs. Some additional background in the introduction and discussion would provide greater context for an international audience, and the methods could be expanded upon for clarity.

Introduction:

- I would provide more background in the introduction on the current landscape of drug use and injection drug use in Nigeria (opioids, stimulants, etc), in the general population and among women. How common is this, and what are the most used drugs in Nigeria (or sub-Saharan Africa)? What is the current landscape of substance use treatment? For example, if opioids are common, is opioid agonist treatment (“medication for opioid use disorder” or “medication assisted treatment”) like methadone or buprenorphine available? Are syringe service programs or needle exchange programs legal? Since this manuscript is focused on women, what are the local laws or policies around substance use during pregnancy? Are they punitive? Understanding this local context is important for interpreting the results/findings of this study.

Methods:

- Some key elements are missing in the methods. I recommend referring to COREQ guidelines as a checklist for comprehensive reporting for qualitative studies. Specific examples include:

o How were the women who inject drugs recruited for the focus group discussions? Supposedly 60 women (6 focus group discussions of 10 women each) were recruited within 3 weeks, according to the methods. This is impressive for a qualitative study, and I am curious how this was accomplished and if it was a representative sample of the region, or all from one clinic?

o How were these women contacted or reached? What was the sampling strategy? How many were contacted vs agreed to participate?

o Inclusion/exclusion criteria?

o Who conducted the interviews? For example, what were their credentials, gender, and training? This is relevant because these participants are being interviewed about a topic that is stigmatized, so their responses may differ if being interviewed by all male interviewers or if there is a component of hierarchy. Did the people conducting the interviews have a relationship with the places where WID were recruited? (I noticed on page 11 that one respondent said “but you people do not charge us”—does that mean the interviewers worked at the facility where the respondent was recruited from?)

o Were participants compensated?

o What language were the interviews conducted in? Are different languages spoken in the different regions, and if so, how was this handled?

o How were data recorded or transcribed? What (if any) software or tools were used to transcribe the interviews?

- Were there any clinical sites or clinicians interviewed as part of this study, and if not, why not? It seems that it would have been helpful to have the perspective of health care workers as well.

Results:

- Basic demographics: Were basic demographic info collected? I would like to have seen a paragraph or table under the characteristics of women who inject drugs that shows the basic demographics of the respondents, particularly the women who inject drugs. Do we know their ages? If HIV status, pregnancy status, marital status were available, those would be pertinent information. Marital status is relevant because of the inclusion of topic of gender based violence in the paper.

- Characteristics of the stakeholders would have been nice to see as well, if available, as it would inform any potential biases in the responses.

- For Methods of Injection section, did we ask where they typically get their needles? (Pharmacy, friends, or needle exchange programs?)

- While reading the results, I could not tell if the WID were all recruited from the OSS clinic and giving feedback on that single program, or describing substance use care in general across Nigeria. It seemed like they were giving feedback about a specific program, and I was surprised to see the generally positive feedback about the substance use care. This is where clarifying the recruitment strategy in the Methods would be helpful. If all the participants were recruited from the same clinic and were interviewed by staff associated with the clinic, responses may be biased.

- On page 9, authors report that respondents from Abia and Oyo seem to be better informed on the matter (of safe injection practices). This is author interpretation, better suited for the discussion, not the results.

- Did the qualitative interviews ask about access to perinatal/antenatal care for WID?

Discussion:

- I was confused about whether the bunk location was where participants wished care was delivered, or if care was currently already being delivered there. Are there health care delivery programs at the bunks?

- I was surprised by the ease of access of clean needles that was reported. Again, it would be helpful to know the current landscape of needle exchange programs in Nigeria.

- I found the inclusion of gender-based violence as part of this qualitative study to be very insightful and profoundly important! Thanks for including this.

- Since opioid use was reported, I wished they had asked more about access to medications for opioid use disorder (e.g. methadone, buprenorphine) since this is the gold standard in many countries for treating opioid use disorder, rather than rehabilitation alone.

- Some of the recommendations proposed in the discussion were not aligned with the study’s own findings. For example, authors mention providing microfinance opportunities to reduce reliance on drug related income, or to cater to the needs of children. I agree with both, however I did not remember seeing these as barriers to care reported in the results section.

Other minor points:

- Several areas of duplication in spelling out women who inject drugs (WID). After defining this acronym in the paper, no need to spell out again.

- I would reduce acronyms where able. If not used frequently in the manuscript, I would just spell out the term and not use the acronym, as it’s difficult to follow for new readers. For example, a lot of acronyms on pages 4-6.

- Page 8 – “WID access healthcare from a range of venues” Is reported in the results. Could authors be more specific about the types of venues where WID access care, in addition to just the bunk?

**********

what does this mean?). If published, this will include your full peer review and any attached files.

**Do you want your identity to be public for this peer review?**  If you choose “no”, your identity will remain anonymous but your review may still be made public.

For information about this choice, including consent withdrawal, please see our Privacy Policy

Reviewer #1: No

**********

[NOTE: If reviewer comments were submitted as an attachment file, they will be attached to this email and accessible via the submission site. Please log into your account, locate the manuscript record, and check for the action link "View Attachments". If this link does not appear, there are no attachment files.]

While revising your submission, please upload your figure files to the Preflight Analysis and Conversion Engine (PACE) digital diagnostic tool, https://pacev2.apexcovantage.com/. PACE helps ensure that figures meet PLOS requirements. To use PACE, you must first register as a user. Registration is free. Then, login and navigate to the UPLOAD tab, where you will find detailed instructions on how to use the tool. If you encounter any issues or have any questions when using PACE, please email PLOS at figures@plos.org. Please note that Supporting Information files do not need this step.

Attachment Submitted filename: PLOS GH women IDU in Nigeria.docx

# Author comment: Author response to Decision Letter 1

**DOI:** 10.1371/journal.pgph.0007008.r002

17 Dec 2025

Attachment Submitted filename: Point by Point Response to reveiwers Manuscript 1.docx

# Peer review: Decision Letter 1

**DOI:** 10.1371/journal.pgph.0007008.r003

**Authors:** Alejandro Torrado Pacheco

3 Mar 2026

PGPH-D-25-01672R1

Understanding Barriers to Harm Reduction Services Among Women who Inject Drugs in Nigeria: a qualitative assessment

PLOS Global Public Health

Dear Dr. Arije,

Thank you for submitting your manuscript to PLOS Global Public Health. After careful consideration, we feel that it has merit but does not fully meet PLOS Global Public Health’s publication criteria as it currently stands. Therefore, we invite you to submit a revised version of the manuscript that addresses the points raised during the review process.

The manuscript has been evaluated by two reviewers, and their comments are available below.

Could you please carefully revise the manuscript to address all comments raised?

Please submit your revised manuscript by Apr 17 2026 11:59PM. If you will need more time than this to complete your revisions, please reply to this message or contact the journal office at globalpubhealth@plos.org. When you're ready to submit your revision, log on to https://www.editorialmanager.com/pgph/ and select the 'Submissions Needing Revision' folder to locate your manuscript file.

Please include the following items when submitting your revised manuscript:

- A letter that responds to each point raised by the editor and reviewer(s). You should upload this letter as a separate file labeled 'Response to Reviewers'.
- A marked-up copy of your manuscript that highlights changes made to the original version. You should upload this as a separate file labeled 'Revised Manuscript with Track Changes'.
- An unmarked version of your revised paper without tracked changes. You should upload this as a separate file labeled 'Manuscript'.

Guidelines for resubmitting your figure files are available below the reviewer comments at the end of this letter.

We look forward to receiving your revised manuscript.

Kind regards,

Alejandro Torrado Pacheco, PhD

PLOS Editor

PLOS Global Public Health

Journal Requirements:

If the reviewer comments include a recommendation to cite specific previously published works, please review and evaluate these publications to determine whether they are relevant and should be cited. There is no requirement to cite these works unless the editor has indicated otherwise.

Additional Editor Comments (if provided):

[Note: HTML markup is below. Please do not edit.]

Reviewers' comments:

Reviewer's Responses to Questions

**Comments to the Author**

Reviewer #1: All comments have been addressed

Reviewer #2: (No Response)

**********

publication criteria?

Reviewer #1: Yes

Reviewer #2: Partly

**********

3. Has the statistical analysis been performed appropriately and rigorously?

Reviewer #1: N/A

Reviewer #2: I don't know

**********

4. Have the authors made all data underlying the findings in their manuscript fully available (please refer to the Data Availability Statement at the start of the manuscript PDF file)?

The PLOS Data policy

Reviewer #1: Yes

Reviewer #2: No

**********

5. Is the manuscript presented in an intelligible fashion and written in standard English?

Reviewer #1: Yes

Reviewer #2: Yes

**********

Reviewer #1: Thank you for the opportunity to review your revised manuscript. This is a qualitative study of women who inject drugs in Nigeria exploring the current landscape of health care access and utilization.

Strengths:

- Important and timely topic on substance use, filling a knowledge and data gap in the literature on SUD and treatment access in sub-Saharan Africa (Nigeria)

- Strong study approach to qualitative studies. Robust interviews covering a wide breadth of topics including access to care, gender-based violence, utilization of syringe services (I reviewed the supplemental documents on their qualitative analysis from the interviews). Appreciated that interviews were conducted among both women who inject drugs and also stakeholders. This reflects somewhat of a human-centered design, bringing together both the end users of a service (the women who inject drugs) and service providers.

- Large sample size for qualitative interviews

Areas of improvement/assessment of the revisions:

- I feel that all of the reviewer comments were adequately addressed in the response sheet as well as in the manuscript text.

Recommending acceptance of manuscript for publication.

Reviewer #2: Review PGPH-D-25-01672_R1

Main comments:

1. There could be some tidying up of the methods to clarify the process for interviews and focus group discussions. I actually suggest separating these two in the Data Collection sub-section and providing rationale for this approach, for example were focus groups conducted to ensure more voices of women who inject drugs were heard?

2. I interpret this as a relatively descriptive qualitative analysis where participants were asked about the domains of interest and then these are presented. If that is incorrect, it might need to be clearer how the themes emerged from the data and what was pre-determined by the interviewer. For example, the aim states “… examine the prevalence of gender-based violence within this population”; however, the Study Instrument description does not detail gender-based violence specifically. Was this emergent from the data? If so, I think this (and the other aims) need finessing to articulate the broad research aim, and the analysis needs to clearly articulate which domains were explored in order to answer this research question.

3. There are a number of spelling and grammatical errors; I have noted some below in the minor comments, but suggest a full proof-read.

4. Given one of the primary utilities of these findings is the availability of certain services for women who inject drugs – I wonder if alongside the results you could include a table indicating the services, if they are available or not, and where they are available? The presentation of these results in qualitative format is less user friendly in this context, and having a simplified/summarised table could be really useful.

5. The perspective of the participants is largely dependent on their position (lived experience or service provision) but in many of the summary statements, it is not clear who the “participants” are. Separating these, I think, is important.

Abstract:

1. Suggest not using acronym WID (see INPUD for guidance on acronyms for key populations: https://inpud.net/words-matter-language-statement-reference-guide/) and generally I suggest use acronyms sparingly (e.g. when the term is used >10 times in the manuscript).

2. The title says “harm reduction access” and the Introduction says “healthcare access”; harm reduction is encompassed within healthcare but not vice versa, I suggest using consistent terminology.

3. Error “ATLAS.ti 24” in last line of Methodology section?

Introduction:

4. First paragraph, sentence 4: I am not sure I agree that blood borne viruses are unique to women who inject drugs, and gender differences differ by country and HIV prevalence among women in Nigeria is approximately 5 times that of men (see PMID: 30726973).

5. The Degenhardt (2013) paper referenced in paragraph 2 does not support this statement. The cited review isn’t about violence, but about preventing HIV for people who inject drugs. It does not describe violence at all.

6. References needed to support statements in paragraph 3.

7. Paragraph 4: typo, “and many face significant barriers”

8. Some of the aims described use language that warrant a quantitative analysis (e.g. “evaluate access”, “examine the prevalence”, “explore patterns”). I suggest editing the aims to more accurately reflect the scope of the analysis. Perhaps removing those quantitative outcomes to simply read explore would work: “This study aims to explore injection drug use among women, understand how they perceive their access to healthcare and social services and understand experiences of gender-based violence.” The current second and fourth aim could be combined for brevity.

Methodology:

9. Typo in section 2.3, second paragraph: currently reads “Incision criteria were Inclusion:” and should perhaps read “Inclusion criteria were:”.

10. ‘IDI’ in Table on pg. 6 not defined. Also Table title needs to be included.

11. To clarify, there were 6 focus groups with women who inject drugs which included 10 women in each? How many non-lived experience stakeholders were recruited?

12. Minor point, but perhaps the box could be brought up under the first paragraph of Study population and Sampling for reference.

13. Who was asked the semi-structured interviews?

14. Suggest a separate sub-heading called “Data analysis”, as paragraphs 3 and 4 do not really fit under “Data collection”.

15. Worth noting when participants were de-identified in the coding process.

Results:

16. To clarify, pregnancy and HIV positivity were not exclusion criteria, this is just to note?

17. I am not trained in qualitative analysis, but I question whether “typologies” is the correct terminology to use here (section 3.2.1)? Alternatively, could you just state that three disctint(?) groups of women who require different things/experience different harm were described by key stakeholders. Were these groups reinforced by the women?

18. Are these typologies mutually exclusive? The second and third are written in such a way that they could conceivably interact.

19. It is difficult to follow perspective – e.g. “Participants reported a wide range of injectable substances used by drug users, especially women.” – I assume this is information gained from the key stakeholders?

20. There is a repeat quote at the end of page 10: “I see OSS as a one-stop shop where I can easily come and make my complaints and take the medication I need and go my way easily (FGD participant/Oyo).” Used above. It does not appear to fit the description provided, is it included by accident?

21. The acronym “OSS” is inconsistently used.

22. Inconsistent use of quotation marks around participant quotes.

23. “Antenatal care services are also accessible..” is included in italics, but I think is part of the non-quote text?

24. Instead of including “XXX” I suggest using block parentheses (e.g. “[hospital name]”) which is more common practice in qualitative research reporting.

25.

**********

what does this mean?). If published, this will include your full peer review and any attached files.

**Do you want your identity to be public for this peer review?**  If you choose “no”, your identity will remain anonymous but your review may still be made public.

For information about this choice, including consent withdrawal, please see our Privacy Policy

Reviewer #1: No

Reviewer #2: No

**********

[NOTE: If reviewer comments were submitted as an attachment file, they will be attached to this email and accessible via the submission site. Please log into your account, locate the manuscript record, and check for the action link "View Attachments". If this link does not appear, there are no attachment files.]

# Author comment: Author response to Decision Letter 2

**DOI:** 10.1371/journal.pgph.0007008.r004

10 Apr 2026

Attachment Submitted filename: Point by Point Response.docx

# Peer review: Decision Letter 2

**DOI:** 10.1371/journal.pgph.0007008.r005

**Authors:** Ilse Bloom

30 Apr 2026

PGPH-D-25-01672R2

Understanding Barriers to Healthcare and Harm Reduction Services Among Women Who Inject Drugs in Nigeria: A Qualitative Assessment

PLOS Global Public Health

Dear Dr. Arije,

Thank you for submitting your manuscript to PLOS Global Public Health. After careful consideration, we feel that it has merit but does not fully meet PLOS Global Public Health’s publication criteria as it currently stands. Therefore, we invite you to submit a revised version of the manuscript that addresses the points raised during the review process.

The manuscript has been further evaluated by two reviewers, and their comments are available below.

The reviewers have raised a number of issues, notably requesting more methodological detail and to ensure that all conclusions presented are supported by the study findings.

Could you please carefully revise the manuscript to address all comments raised?

Please submit your revised manuscript by Jun 29 2026 11:59PM. If you will need more time than this to complete your revisions, please reply to this message or contact the journal office at globalpubhealth@plos.org. When you're ready to submit your revision, log on to https://www.editorialmanager.com/pgph/ and select the 'Submissions Needing Revision' folder to locate your manuscript file.

Please include the following items when submitting your revised manuscript:

- A letter that responds to each point raised by the editor and reviewer(s). You should upload this letter as a separate file labeled 'Response to Reviewers'.
- A marked-up copy of your manuscript that highlights changes made to the original version. You should upload this as a separate file labeled 'Revised Manuscript with Track Changes'.
- An unmarked version of your revised paper without tracked changes. You should upload this as a separate file labeled 'Manuscript'.

Guidelines for resubmitting your figure files are available below the reviewer comments at the end of this letter.

As the corresponding author, your ORCID iD is verified in the submission system and will appear in the published article. PLOS supports the use of ORCID, and we encourage all coauthors to register for an ORCID iD and use it as well. Please encourage your coauthors to verify their ORCID iD within the submission system before final acceptance, as unverified ORCID iDs will not appear in the published article. *Only*  the individual author can complete the verification step; PLOS staff *cannot*  verify ORCID iDs on behalf of authors.

We look forward to receiving your revised manuscript.

Kind regards,

Ilse Bloom

Staff Editor

PLOS Global Public Health

**Journal Requirements:**

If the reviewer comments include a recommendation to cite specific previously published works, please review and evaluate these publications to determine whether they are relevant and should be cited. There is no requirement to cite these works unless the editor has indicated otherwise.

**Additional Editor Comments (if provided):**

[Note: HTML markup is below. Please do not edit.]

Reviewers' comments:

Reviewer's Responses to Questions

**Comments to the Author**

Reviewer #1: All comments have been addressed

Reviewer #2: All comments have been addressed

**********

publication criteria?

Reviewer #1: Yes

Reviewer #2: Yes

**********

3. Has the statistical analysis been performed appropriately and rigorously?

Reviewer #1: N/A

Reviewer #2: Yes

**********

4. Have the authors made all data underlying the findings in their manuscript fully available (please refer to the Data Availability Statement at the start of the manuscript PDF file)?

The PLOS Data policy

Reviewer #1: Yes

Reviewer #2: No

**********

5. Is the manuscript presented in an intelligible fashion and written in standard English?

Reviewer #1: Yes

Reviewer #2: Yes

**********

**Reviewer #1:**  Thank you for the opportunity to read this revised manuscript on this qualitative study of Nigerian women who inject drugs and their access to healthcare and harm reduction services. I appreciate the authors making thoughtful and detailed changes in response to the prior feedback. Overall, the manuscript is stronger and clearer with these changes and fulfills a literature gap in understanding barriers to harm reduction services among women in Nigeria. However, there are still a number of misspellings and misuse of abbreviations throughout the manuscript which need to be corrected before final publication, should the manuscript be accepted and published.

Abstract:

- STI, PMTCT, and WID need to be defined

- “Availability varied by service type and location” is vague. If there were specific patterns that emerged (e.g. a primary service type or location), state them.

- The term “bunks” is not universally known, although I acknowledge this is defined later in the manuscript and the abstract may not have the space to define this

- In the conclusion, ‘gender-responsive healthcare’ and ‘safer referral pathways’ are vague. Could you be more specific about what the call to action is here in this context and population? (e.g. integration of reproductive health services, trauma-informed care, legal protection, etc?)

Introduction:

- 3rd paragraph: please replace “drug users” with people who use drugs, which is person-first language and less stigmatizing

- 3rd paragraph: please replace “drug abuse” with prescription drug misuse

- In general, unless an abbreviation is used frequently throughout the manuscript, there is no need to include the abbreviation. For example, intimate partner violence (IPV) and opioid substitution therapy (OST) are only used once. No need to abbreviate these.

- You use opioid substitution therapy and the opioid agonist treatment in the next sentence, which are referring to the same treatments. You can just use opioid agonist treatment, or preferred would be “medications for opioid use disorder” which is slightly more accurate, since buprenorphine is a partial agonist. I would change to medications for opioid use disorder, which you use later in the discussion.

- PWID is defined a second time in the 5th paragraph of the intro

Methods:

- In general, if an abbreviation is not used frequently, please eliminate the abbreviation and just use the whole name. It is confusing to try to read and keep track of so many abbreviations. I counted at least 10 abbreviations in the first few paragraphs of the methods section. The only terms justified using abbreviations in my opinion in this section would be PWID, WID, and FGD.

- Focus group discussions (FGD) is defined multiple times

- Spelling error: 'enrolment' on page 6

- Were the WID who were interviewed compensated for their participation?

- Typo/error in first 2 sentences under Data Collection on page 7

- How was it determined when sufficient # of interviews were completed? Was there an aim to reach thematic saturation? Was it reached?

Results:

- 3.1.1 Background characteristics of study participants- since this is not reported as a result, I think this section belongs in the Methods section

- Women who inject drugs (WID) is defined earlier but spelled out in various places throughout the document. I would do a search of the document to make sure all areas of “women who inject drugs” are replaced with WID unless it is starting a sentence

- "interveiwed” typo on page 8

- Really like the addition of Table 2 to this revised manuscript

- Replace 3.6 title “some recommendations” to something more descriptive. For example, “Potential Facilitators to Improving Healthcare Access” or “Suggestions for Improving Healthcare Access for WID”

Discussion:

- The 2nd paragraph in the discussion on bunks being preferred location of service delivery is very interesting as you mention. This method of service delivery is often known as decentralized service delivery, and/or you could mention how this study reflects the growing interest in “differentiated service delivery models.” The findings you are describing are essentially differentiated service delivery models, which you can mention and cite.

- You note that even though harm reduction services were available, there was observed persistence in high-risk behaviors like needle sharing. Do you think this is because of the lack of universal provision of harm reduction services or do you think there is another barrier, like knowledge gaps on safe injection?

- Limitation section– typo, ‘on-gong’

Conclusion:

- What do you mean by “international narcotics”?

- I think you can explicitly state that the preference for accessing health services in informal bunk settings suggests using differentiated service delivery models to meet the needs of WID

- I am not sure I agree with the need for rehabilitation centers as the main conclusion of this study. It was mentioned by stakeholders but not the WID themselves. The data on efficacy of inpatient/intensive rehab vs outpatient treatment for substance use disorders in nuanced in the literature. For example, for opioid use disorder, outpatient treatment with medications for opioid use disorder have been shown to have better outcomes than inpatient/residential treatment or detoxification programs. I think the data for other substances is less clear. I think it’s notable that your stakeholders highlighted the absence of rehab centers, but your participants/WID did not mention this as a desired need or gap. What I do agree with and find interesting is a need for more tailored, gender-sensitive programs within rehab centers, such as women-only programs. While it’s appropriate to leave in the results and discussion, I feel less compelled for this to be one of the main takeaways in the conclusion based on the stakeholder opinion alone, when this was not a main finding from the WID perspective.

**Reviewer #2:**  Thank you for addressing my previous comments, the manuscript is substantially improved. There are only very minor things to consider:

1. Second sentence in first paragraph under 2.5 Data Collection should be removed.

2. Please include definitions for acronyms in a table note. The tables should be interpretable on their own.

3. I suggested using acronyms sparingly throughout the manuscript, including not using ‘WID’ – but this has not been done and only partly done in the abstract. There are now some undefined acronyms in the abstract (including WID and PMTCT) and throughout the paper. There are acronyms that are only used once and never again (e.g. OST), this is unnecessary.

**********

what does this mean?). If published, this will include your full peer review and any attached files.

**Do you want your identity to be public for this peer review?**  If you choose “no”, your identity will remain anonymous but your review may still be made public.

For information about this choice, including consent withdrawal, please see our Privacy Policy

Reviewer #1: No

Reviewer #2: No

**********

[NOTE: If reviewer comments were submitted as an attachment file, they will be attached to this email and accessible via the submission site. Please log into your account, locate the manuscript record, and check for the action link "View Attachments". If this link does not appear, there are no attachment files.]

**Figure Resubmissions:**

# Author comment: Author response to Decision Letter 3

**DOI:** 10.1371/journal.pgph.0007008.r006

1 Jun 2026

Attachment Submitted filename: Response to reviewers.docx

# Editor report: Decision Letter 3

**DOI:** 10.1371/journal.pgph.0007008.r007

**Authors:** Alejandro Torrado Pacheco

30 Jun 2026

PGPH-D-25-01672R3

Understanding Barriers to Healthcare and Harm Reduction Services Among Women Who Inject Drugs in Nigeria: A Qualitative Assessment

PLOS Global Public Health

Dear Dr. Arije,

Thank you for submitting your manuscript to PLOS Global Public Health. After careful consideration, we feel that it has merit but does not fully meet PLOS Global Public Health’s publication criteria as it currently stands. Therefore, we invite you to submit a revised version of the manuscript that addresses the points raised during the review process.

Thank you for addressing the reviewer's comments. There are two points that require attention still. Could you please address the following?

i) Please address Reviewer 1's comment about data saturation. The statement currently in the manuscript does not provide any details on how saturation was assessed or whether it was reached.

ii) Please revisit the Reviewer's comments about the section in the Conclusions regarding rehabilitation centers. As the Reviewer notes, this is not reflected in WID interviews. I suggest a more nuanced discussion of this point reflecting the difference in the views of stakeholders vs WID.

Please submit your revised manuscript by Jul 30 2026 11:59PM. If you will need more time than this to complete your revisions, please reply to this message or contact the journal office at globalpubhealth@plos.org. When you're ready to submit your revision, log on to https://www.editorialmanager.com/pgph/ and select the 'Submissions Needing Revision' folder to locate your manuscript file.

Please include the following items when submitting your revised manuscript:

- A letter that responds to each point raised by the editor and reviewer(s). You should upload this letter as a separate file labeled 'Response to Reviewers'.
- A marked-up copy of your manuscript that highlights changes made to the original version. You should upload this as a separate file labeled 'Revised Manuscript with Track Changes'.
- An unmarked version of your revised paper without tracked changes. You should upload this as a separate file labeled 'Manuscript'.

Guidelines for resubmitting your figure files are available below the reviewer comments at the end of this letter.

As the corresponding author, your ORCID iD is verified in the submission system and will appear in the published article. PLOS supports the use of ORCID, and we encourage all coauthors to register for an ORCID iD and use it as well. Please encourage your coauthors to verify their ORCID iD within the submission system before final acceptance, as unverified ORCID iDs will not appear in the published article. *Only*  the individual author can complete the verification step; PLOS staff *cannot*  verify ORCID iDs on behalf of authors.

We look forward to receiving your revised manuscript.

Kind regards,

Alejandro Torrado Pacheco, PhD

PLOS Editor

PLOS Global Public Health

**Journal Requirements:**

If the reviewer comments include a recommendation to cite specific previously published works, please review and evaluate these publications to determine whether they are relevant and should be cited. There is no requirement to cite these works unless the editor has indicated otherwise.

Please review your reference list to ensure that it is complete and correct. If you have cited papers that have been retracted, please include the rationale for doing so in the manuscript text, or remove these references and replace them with relevant current references. Any changes to the reference list should be mentioned in the rebuttal letter that accompanies your revised manuscript. If you need to cite a retracted article, indicate the article’s retracted status in the References list and also include a citation and full reference for the retraction notice.

**Additional Editor Comments (if provided):**

[Note: HTML markup is below. Please do not edit.]

**Reviewers' comments:**

[NOTE: If reviewer comments were submitted as an attachment file, they will be attached to this email and accessible via the submission site. Please log into your account, locate the manuscript record, and check for the action link "View Attachments". If this link does not appear, there are no attachment files.]

**Figure Resubmissions:**

# Author comment: Author response to Decision Letter 4

**DOI:** 10.1371/journal.pgph.0007008.r008

11 Jul 2026

Attachment Submitted filename: Response to reviewers_.docx

# Editor report: Decision Letter 4

**DOI:** 10.1371/journal.pgph.0007008.r009

**Authors:** Julia Robinson

19 Jul 2026

Understanding Barriers to Healthcare and Harm Reduction Services Among Women Who Inject Drugs in Nigeria: A Qualitative Assessment

PGPH-D-25-01672R4

Dear Dr. Arije,

We are pleased to inform you that your manuscript 'Understanding Barriers to Healthcare and Harm Reduction Services Among Women Who Inject Drugs in Nigeria: A Qualitative Assessment' has been provisionally accepted for publication in PLOS Global Public Health.

Before your manuscript can be formally accepted you will need to complete some formatting changes, which you will receive in a follow up email. A member of our team will be in touch with a set of requests.

Please note that your manuscript will not be scheduled for publication until you have made the required changes, so a swift response is appreciated.

IMPORTANT: The editorial review process is now complete. PLOS will only permit corrections to spelling, formatting or significant scientific errors from this point onwards. Requests for major changes, or any which affect the scientific understanding of your work, will cause delays to the publication date of your manuscript.

If your institution or institutions have a press office, please notify them about your upcoming paper to help maximize its impact. If they'll be preparing press materials, please inform our press team as soon as possible -- no later than 48 hours after receiving the formal acceptance. Your manuscript will remain under strict press embargo until 2 pm Eastern Time on the date of publication. For more information, please contact globalpubhealth@plos.org.

Thank you again for supporting Open Access publishing; we are looking forward to publishing your work in PLOS Global Public Health.

Best regards,

Julia Robinson

Executive Editor

PLOS Global Public Health

***********************************************************

Reviewer Comments (if any, and for reference):
